# Correction: *Management of patients with an advance decision and suicidal behaviour: a systematic review*

**DOI:** 10.1136/bmjopen-2018-023978corr1

**Published:** 2019-05-05

Nowland R, Steeg S, Quinlivan LM, *et al*. Management of patients with an advance decision and suicidal behaviour: a systematic review. *BMJ Open* 2019;**9:**e023978. doi: 10.1136/bmjopen-2018-023978

This article was previously published with an error.

In second affiliation, NHIR should be NIHR. The correct affiliation number 2 is:

NIHR Greater Manchester Patient Safety Translational Research Centre, University of Manchester, UK

