# Supplementary material for: Management of patients with an advance decision and suicidal behaviour: a systematic review
Source: BMJ Open. 2019 Mar 13;9(3):e023978. doi: 10.1136/bmjopen-2018-023978 (PMC6429970; doi:10.1136/bmjopen-2018-023978)
Supplement: Supplementary data [file bmjopen-2018-023978supp001.pdf]

Supplementary Information 1: Database Search Strategy

Psychinfo:

(((advance decision or advance directive or advance statement or living will or living wills or mental health directive or Ulysses contract or Ulysses contracts or psychiatric will or psychiatric wills or mental competency or mental capacity or healthcare power of attorney or health care power of attorney or antecedent decision or antecedent wish* or preemptive suicide or antecedent refusal or resuscitation orders)

and

(suicide or attempted suicide or self mutilation or self-harm or deliberate self-harm or self harm or parasuicid$ or para-suicd$ or "self-injurious behaviour" or drug overdose or self immolation or self poisoning or self-destructive behav or autoaggress$ or automutilia$)) not (euthanasia or assisted suicide)).af.

Pubmed:

("advance decisions" or "advance directives" or "advance statement" or "living will" or "living wills" or "mental health directive" or "ulysses contract" or "ulysses contracts" or "psychiatric will" or "psychiatric wills" or "mental competency" or "mental capacity" or "healthcare power attorney" or "healthcare power of attorney" or "antecedent decision" or "antecedent wish" or "preemptive suicide" or "antecedent refusal" or "resuscitation orders" or "do not resuscitate" or DNR order)
suicid

EBESCO:

(suicide or attempted suicide or self mutilation or self-harm or deliberate self-harm or self harm or parasuicid$ or para-suicd$ or "self-injurious behaviour" or drug overdose or self immolation or self poisoning or self-destructive behav or autoaggress$ or automutilia$).ab

and

(advance decision or advance directive or advance statement or living will or living wills or mental health directive or Ulysses contract or Ulysses contracts or psychiatric will or psychiatric wills or mental competency or mental capacity or healthcare power of attorney or health care power of attorney or antecedent decision or antecedent wish* or preemptive suicide or antecedent refusal or resuscitation orders).ab

EMBASE:

(advance decision or advance directive or advance statement or living will or living wills or mental health directive or Ulysses contract or Ulysses contracts or psychiatric will or psychiatric wills or mental competency or mental capacity or healthcare power of attorney or health care power of attorney or antecedent decision or antecedent wish* or preemptive suicide or antecedent refusal or resuscitation orders).ab.

and

(suicide or attempted suicide or self mutilation or self-harm or deliberate self-harm or self harm or parasuicid$ or para-suicd$ or "self-injurious behaviour" or drug overdose or self immolation or self poisoning or self-destructive behav or autoaggress$ or automutilia$).ab

MEDLINE:

(((advance decision or advance directive or advance statement or living will or living wills or mental health directive or Ulysses contract or Ulysses contracts or psychiatric will or psychiatric wills or mental competency or mental capacity or healthcare power of attorney or health care power of attorney or antecedent decision or antecedent wish* or preemptive suicide or antecedent refusal or resuscitation orders) and (suicide or attempted suicide or self mutilation or self-harm or deliberate self-harm or self harm or parasuicid$ or para-suicd$ or "self-injurious behaviour" or drug overdose or self immolation or self poisoning or self-destructive behav or autoaggress$ or automutilia$)) not (euthanasia and assisted suicide)).ab.

CINAHL

AB (advance decisions OR (advance directives and living wills) OR mental capacity OR mental competency OR health care power of attorney OR antecedent decision OR preemptive suicide OR resuscitation orders OR (dnr or do not resuscitate) OR (dnr orders and ethical principles))

AND AB suicide OR suicide attempt OR self-harm OR self harm OR deliberate self harm OR self-injurious behavior OR (self injury or self harm or self mutilation) OR drug overdose OR self immolation OR self-destructiv behaviors OR self-poisoning
AB ((advance decisions OR (advance directives and living wills) OR mental capacity OR mental competency OR health care power of attorney OR antecedent decision OR preemptive suicide OR resuscitation orders OR (dnr or do not resuscitate) OR (dnr orders and ethical principles))) AND AB (suicide OR suicide attempt OR self-harm OR self harm OR deliberate self harm OR self-injurious behavior OR (self injury or self harm or self mutilation) OR drug overdose OR self immolation OR self-destructiv behaviors OR self-poisoning) NOT AB assisted suicide NOT AB (euthanasia or assisted suicide)

Social Policy and Practice:

(("advance decisions" or "advance directives" or "advance statement" or "living will" or "living wills" or "mental health directive" or "ulysses contract" or "ulysses contracts" or "psychiatric will" or "psychiatric wills" or "mental competency" or "healthcare power attorney" or "healthcare power of attorney" or "antecedent decision" or "antecedent wish" or "pre emptive suicide" or "preemptive suicide" or "antecedent refusal" or "resuscitation orders" or "do not resuscitate" or "DNR order") not (euthanasia and "assisted suicide")).af.

and

(suicide or "attempted suicide" or "self-mutilation" or "deliberate self-harm" or "self-harm" or Parasuicide or Suicid* or "drug-overdose" or "self-poisioning" or "self-immolation" or "suicidal behav*" or "self-destructive behav*" or Autoaggress$ or "self-injurious behav*" or "non suicidal self-injury" or "non fatal self-harm" or "completed suicide" or automutilla$).af
