# Supplementary material for: Management of patients with an advance decision and suicidal behaviour: a systematic review
Source: BMJ Open. 2019 Mar 13;9(3):e023978. doi: 10.1136/bmjopen-2018-023978 (PMC6429970; doi:10.1136/bmjopen-2018-023978)
Supplement: Supplementary data [file bmjopen-2018-023978supp002.pdf]

Supplementary Information 2: Quality Assessment of studies

	Question 1		Question 2		Question 3		Question 4		Question 5		Methodological quality
Author	Yes	No	Yes	No	Yes	No	Yes	No	Yes	No	
Bryne[2]		X		X		X		X		X	Poor
Callaghan & Ryan[22]		X	X		X		X		X		Moderate
Chalfin et al[23]		X	X			X	X		X		Poor
Cook et al[24]		X	X		X		X		X		Moderate
Dresser[6]		X		X		X	X			X	Poor
David et al[25]		X	X		X		X		X		Moderate
Frank[7]		X	X			X		X	X		Poor
Kapur et al[8]		X	X		X			X		X	Poor
Mitchell[26]		X		X		X		X		X	Poor
Muzaffer[27]		X	X			X	X			X	Poor
Richardson[28]		X	X		X		X		X		Moderate
Ryan & Callaghan[29]		X	X		X		X		X		Moderate
Sontheimer[30]		X	X		X			X	X		Poor
Szawarski[31]		X	X		X		X		X		Moderate
Volpe et al[32]		X	X			X		X		X	Poor

Note: *Selection*: question 1: Did the patient(s) represent the whole experience of the investigator or is the selection method unclear to the extent that other patients with similar presentations may not have been presented?; *Ascertainment*: question 2: Was the case adequately ascertained?, question 3: Was the outcome adequately ascertained?; *Causality*: question 4: Was follow-up long enough for outcomes to occur?; *Reporting*: question 5: Is the case described with sufficient details to allow practitioners to make inferences on their own practice?
