# Supplementary material for: Management of patients with an advance decision and suicidal behaviour: a systematic review
Source: BMJ Open. 2019 Mar 13;9(3):e023978. doi: 10.1136/bmjopen-2018-023978 (PMC6429970; doi:10.1136/bmjopen-2018-023978)
Supplement: Author's manuscript [file bmjopen-2018-023978.draft_revisions.pdf]

BMJ Open is committed to open peer review. As part of this commitment we make the peer review history of every article we publish publicly available.

When an article is published we post the peer reviewers' comments and the authors' responses online. We also post the versions of the paper that were used during peer review. These are the versions that the peer review comments apply to.

The versions of the paper that follow are the versions that were submitted during the peer review process. They are not the versions of record or the final published versions. They should not be cited or distributed as the published version of this manuscript.

BMJ Open is an open access journal and the full, final, typeset and author-corrected version of record of the manuscript is available on our site with no access controls, subscription charges or pay-per-view fees (http://bmjopen.bmj.com).

If you have any questions on BMJ Open's open peer review process please email info.bmjopen@bmj.com

BMJ Open

The management of patients with an advance decision and suicidal behaviour: A systematic review

Journal:	*BMJ Open*
Manuscript ID	bmjopen-2018-023978
Article Type:	Research
Date Submitted by the Author:	10-May-2018

Complete List of Authors:	Nowland (Harris), Rebecca; University of Manchester, Centre for Health and safety<br>Steeg, Sarah; University of Manchester, Psychiatry<br>Quinlivan, Leah; University of Manchester , Brain, Behaviour and Mental Health<br>Cooper, Jayne; University of Manchester,<br>Huxtable, Richard; University of Bristol, School of Social and Community Medicine<br>Hawton, Keith; Centre for Suicide Research, Oxford University, Psychiatry<br>Gunnell, DJ; University of Bristol, Social Medicine<br>Allen, Neil; University of Manchester, School of Law<br>Mackway-Jones, Kevin; Manchester Royal Infirmary, Emergency Department<br>Kapur, Navneet; University of Manchester, Centre for suicide prevention

Keywords:	Suicide & self-harm < PSYCHIATRY, suicidal behaviour, advance decisions, advance directives, living wills

SCHOLARONE™
Manuscripts

Title: The management of patients with an advance decision and suicidal behaviour: A systematic review

Author list: Rebecca Nowland, PhD[1]; Sarah Steeg, PhD[1]; Leah Quinlivan, PhD[1,2]; Jayne Cooper, PhD[1], Prof Richard Huxtable[3]; Prof Keith Hawton, FMedSc[4]; Prof David Gunnell DSc[5], Neil Allen (Barrister)[6], Prof Kevin Mackway-Jones[7], Prof Nav Kapur, FRPsych[1,2, 8]

1. Centre for Suicide Prevention, Manchester Academic Health Science Centre, University of Manchester, England
2. NIHR Greater Manchester Patient Safety Translational Research Centre, England
3. Centre for Ethics in Medicine, University of Bristol, England
4. Centre for Suicide Research, University Department of Psychiatry, Warneford hospital, Oxford, England
5. Department of Population Health Sciences, University of Bristol, England
6. School of Law, University of Manchester, England
7. Emergency Department, Manchester Royal Infirmary, Manchester University NHS Foundation Trust, Manchester, England
8. Greater Manchester Mental Health NHS Foundation Trust, Manchester, England.

Corresponding author: Dr Rebecca Nowland

Address: Jean McFarlane Building, University of Manchester, Oxford Road, Manchester M13 9PL

Telephone: +44 (0)161 275 0727

Email: Rebecca.Nowland@manchester.ac.uk

Word count = 5662 (excluding abstract and references)

For peer review only

Authorship and contribution

All authors made substantial contributions to the study. RN and LQ conducted the initial scoping search. RN designed the review and data extraction/analysis with input from NK and SS. RN and SS screened and reviewed the articles and performed data extraction/analysis, interpreted the results and wrote the first draft. All authors contributed to subsequent drafts and approved the final version. All authors take responsibility for the integrity of the data analysis. NK is the guarantor of the study.

Declaration of Interest

DG, KH, and NK are members of the Department of Health's (England) National Suicide Prevention Advisory Group. NK chaired the NICE guideline development group for the longer term management of self-harm and the NICE Topic Expert Group (which developed the quality standards for self-harm services). He is currently chair of the updated NICE guideline for Depression. KH and DG are NIHR Senior Investigators. KH is also supported by the Oxford Health NHS Foundation Trust and NK by the Greater Manchester Mental Health NHS Foundation Trust.

Funding

This paper presents independent research funded by the National Institute of Health Research (NIHR) under its Programme Grants for Applied Research Programme (Grant Reference Number RP-PG-0610-10026). The views expressed are those of the authors and not necessarily those of the NHS, the National Institute of Health Research or the Department of Health.

Data sharing statement

No additional data are available

Provenance and peer review

Not commissioned; externally peer reviewed

For peer review only

Acknowledgements

We would to thank Rosie Davies, Research Fellow in Patient and Public Involvement at the University of Bristol, for her input into the study.

For peer review only

ABSTRACT

Objectives: To synthesise existing literature on the management of advance decisions and suicidal behaviour.

Design: A systematic search of 7 bibliographic databases was conducted to identify studies relating to advance decisions and suicidal behaviour. Studies on terminal illness or end of life care were excluded to focus on the use of advance decisions in the context of suicidal behaviour. A textual synthesis of data was conducted and themes were identified by using an adapted thematic framework analysis approach.

Results: Overall 634 articles were identified, of which 35 were retained for full text screening. Fifteen relevant articles were identified following screening. Those articles pertained to actual clinical cases or fictional scenarios. Clinical practice and rationale for management decisions varied. Five themes were identified: 1) tension between patient autonomy and protecting a vulnerable person, 2) appropriateness of advance decisions for suicidal behaviour, 3) uncertainty about the application of legislation, 4) the length of time needed to consider all the evidence vs. rapid decision-making for treatment, and 5) importance of seeking support and sharing decision-making.

Conclusions: Advance decisions present particular challenges for clinicians when associated with suicidal behaviour. Recommendations for practice and supervision for clinicians may help to reduce the variation in clinical practice.

Keywords: self-harm, suicidal behaviour, advance directives, advance decisions, living wills, Ulysses directives

For peer review only

Article Summary

Article focus

- Management of advance decisions in the context of suicidal behaviour is understudied
- Awareness of the challenges related to this particular presentation of advance decision will guide policy and practice

Key messages

- Managing advance decisions in the context of suicidal behaviour is challenging
- There is variability in practice and rationale behind clinical decisions
- Taking time to consider all the evidence, consulting fully with mental health clinicians and seeking legal and/or ethical advice may help with some of these challenges
- Support of a relevant healthcare professional at the time of writing the advance decision may also be useful

Strengths and limitations of this study

- Timely systematic review considering the challenges relating to advance decisions in the context of suicidal behaviour
- Review involves journal articles from a variety of countries from a range of different disciplines
- Paucity of evidence for this specific presentation of advance decision

For peer review only

INTRODUCTION

An advance decision is typically a written document that outlines a person's desire to refuse certain treatments, including life-saving treatment, when there is a potential for a person to lose the mental capacity to make treatment decisions in the future (Mental Capacity Act 2005). In order for an advance decision to be valid, the person must have mental capacity at the time of writing the document. Mental capacity is defined as the ability to make a decision and involves understanding and weighing information relating to a decision and alternative options and retaining that information long enough to make the decision (Mental Capacity Act 2005). The Mental Capacity Act in England and Wales refers to "advance decisions to refuse treatment (ADRT)," but more widely these documents are referred to as "advance directives" and/or "living wills". We use "advance decision" throughout in this paper to refer to written documents stating a refusal of treatment made in advance.

There are important cross-national variations in legislation; in some countries, the use of advance decisions is not permitted (i.e. Turkey, Japan), while in others, advance decisions are legislated for (i.e. the UK and US). The UK, Australia and US have similar legal standards with some state-wide variation in the US and Australia (Byrne, 2002), with some states adopting the common law right to make an advance decision and others allowing the use of a surrogate or proxy decision maker (i.e. to make healthcare decisions on behalf of the patient). There is also considerable variation in practice between countries where advance decisions are permitted. For example, in Germany, advance decisions are recognised but require court approval in each case (Byrne, 2002).

Advance care planning and advance decisions for psychiatric care are becoming more common and have some potential benefits, including enhancing patient autonomy and engagement, promoting adherence to treatment plans, improving continuity of care with fewer psychiatric admissions, reducing the use of social workers' time and lower levels of violent acts (Campbell & Kisley, 2012; Swanson et al., 2000). However, concerns have been raised about clinical management of advance decisions in the particular context of suicidal behaviour (e.g. Dresser, 2010; Frank, 2013). Existing literature, from a variety of academic

For peer review only

and clinical perspectives, suggests there is little consistency in practice and there are specific challenges with advance decisions and suicidal behaviour. Such scenarios raise questions about whether a person with a wish to end their life has the capacity to make that decision or if their capacity is affected by mental illness, and whether an advance decision is appropriate in this particular context (Kapur et al., 2010).

The management of suicidal behaviour is a significant challenge for clinicians in the emergency services. Each year over 200,000 people present to emergency departments in England with self-harm (Hawton et al., 2007), with 16% of those presenting to hospital with a repeat self-harm episode within a year (Carroll et al., 2014). In a recent study, in three out of 121 fatal cases of self-poisoning in 2005, the patients had an advance decision (Kapur et al., 2010). Given that patient autonomy is encouraged in modern healthcare and is assuming greater prominence, it is likely that the number of advance decisions relating to suicidal behaviour will grow.

Rationale

While literature reviews of advance decisions, both more broadly and specific to advance decisions and/or advance care planning relating to "end of life" care exist (e.g. Houben et al., 2014; Brinkman-Stoppelenburg, Rietjens & Van der Heide, 2014), there are no reviews on the management of advance decisions in the context of suicidal behaviour when the patient does not have a chronic or terminal physical illness. Despite the legislative context being similar for end of life care, the ethical considerations, emotional challenges and clinical decision-making may be different for suicidal behaviour without a chronic or terminal physical illness.

Aim

To systematically review and synthesise literature on the treatment and clinical management of patients presenting with an advance decision in the context of suicidal behaviour without a chronic or terminal physical illness.

For peer review only

Method

The review was conducted in accordance with PRISMA guidelines (Moher et al., 2015; Shamseer et al., 2015) and guidance for conducting narrative synthesis in healthcare (CRD, 2009). There is no protocol for the review. We used the PRISMA checklist when writing our report (Moher et al., 2015).

Search strategy and data sources

Content experts and clinical practitioners on the research team assisted with compiling key words and/or phrases (see Table 1) and conducting an electronic search of six databases (EMBASE, MEDLINE, PSYCHINFO, Social Policy and Practice, CINAHL and Medline). A full electronic search was also conducted on WestLaw (an online library of UK legal information) using the following search terms: *advance decisions, advance directives* AND *wills, suicide*. In addition, the reference sections of all included sources were consulted and authors' personal files were also searched to ensure that potentially eligible sources were not omitted. No study design, date or language restrictions were imposed.

For peer review only

Table 1. Search terms for each topic

Advance directives	*OR*	Mental capacity	*AND*	Suicidal behaviour
advance decisions		mental		Suicide
advance directives		competency		attempted suicide
advance statement		mental capacity		self-mutilation
living will(s)				self-harm
mental health directive				deliberate self-harm
Ulysses contract(s)				parasuicide
psychiatric will(s)				self-injurious behaviour
antecedent decision/wish				drug overdose
pre-emptive suicide				self-immolation
antecedent refusal				self-poisoning
resuscitation order				self-destructive behaviour
health care power of attorney				auto aggression
				automutilation

Literature searches were conducted during the period April 2016 to July 2017. The specific inclusion and exclusion criteria are detailed in Table 2.

Table 2. Inclusion and Exclusion criteria

Inclusion	Exclusion
Literature on medical management and/or medico legal and/or ethical issues relating to people who present to hospital with advance decisions* with pre-existing mental health issues (also include Do Not Resuscitate orders, DNRs) in the context of suicidal behaviour or self-harm	Literature on medical management of people who present to hospital with advance decisions but with primary conditions which were not mental health related e.g. HIV/AIDS, chronic physical health conditions or disabilities, neurodegenerative diseases and/or specific patient groups e.g. mother/baby.
Literature relating patients over the age of 18 years	Literature relating to medical management of euthanasia, assisted suicide, end of life, wills/inheritance (i.e. monetary or property issues)
**Documents included:**<br>Opinion and review articles<br>Case studies<br>Empirical studies/surveys	**Documents excluded:**<br>Book review<br>Reponses to articles

* or other terms such as *advance decisions, advance directives, advance statement, living will(s), mental health directive, Ulysses contract(s), psychiatric will(s), mental competency, mental capacity, health care power of attorney, antecedent decision/wish, pre-emptive suicide, antecedent refusal, resuscitation order* or living will, advance directive, Ulysses contract

For peer review only

Study selection

Titles and abstracts were screened, with a random sample of 10% of the articles independently screened by another researcher. Additional information was sought where there were any disagreements, which were then resolved through discussion. An acceptable concordance rate between the inclusion decisions was predefined as agreement on at least 90% of the articles, which was achieved for screening on title and abstract. Full text screening of the selected articles was conducted by two researchers independently, with full agreement being achieved at this stage.

Data extraction and analysis

A preliminary analysis of the data was conducted (CRD, 2009). Studies were from a range of disciplines and involved reviews of clinical cases or fictional scenarios. It was deemed appropriate to conduct a narrative synthesis because this particular approach is useful when synthesising textual findings from diverse literatures (CRD, 2009). Narrative synthesis was conducted in two phases: 1) a *textual synthesis*, and 2) an adapted *thematic framework analysis* (Richie & Spencer, 1994).

First, the *textual synthesis* of the data was conducted by extracting key factual information from each study and details of the case studies. The information was then summarised and tabulated to map the literature that cited the same clinical case. Data extraction and summarisation was completed independently by two researchers using a pre-determined data extraction sheet.

Second, an adapted *thematic framework analysis* approach (Richie & Spencer, 1994) was used to examine key themes discussed in the selected papers. This involved five stages: initial open coding, indexing, descriptive summaries, charting and tabulation and interpretation. *Initial open coding* generated three general categories representing the most discussed issues across the selected articles: 1) key issues with an advance decision relating

For peer review only - http://bmjopen.bmj.com/site/about/guidelines.xhtml

to suicidal behaviour, 2) challenges in clinical decision-making for advance decisions relating to suicidal behaviour, and 3) recommendations for practice. These three categories were used to index the data and as a framework to extract and summarise data. Extracted data was then used to form *descriptive summaries*. Indexing, extracting and summarising were conducted independently by two researchers. Resulting summaries were compared and discussions were held to clarify any differences. *Charting and tabulation* was conducted by charting the summaries by discipline. *Interpretation* of the data was conducted by thematic analysis of the summary charts to highlight the main recurrent and most important themes (CRD, 2009). Two researchers conducted the thematic analysis independently and then discussed and finalised themes. Saturation of the themes was established when no further themes emerged and could not be further collapsed. “Vote counting” was used to identify the frequency with which the themes appeared in the selected papers (Ryan, 2013).

Quality assessment

The papers mostly comprised accounts of clinical cases written by clinicians and ethical or legal experts. We used recommended criteria to assess the quality of case reports (Murad, Sultan, Haffar, & Bazerbachi, 2018), including considering the extent to which the patient experience represented other possible cases and the clarity and detail with which the case and the outcome was described. We also considered the reflexivity of the author/s, their expertise and how they were involved in the clinical case (for example as a clinician or legal/ethics consultant). Authors of the papers reflected on the management of the clinical case, rationale for decision made and issues relating to advance decisions and suicidal behaviour more generally. Data in all selected papers was considered by the research team to be of sufficient depth and quality for analysis and informative for practice.

Patient and Public Involvement

The study is a review of literature so there was no patient involvement in the design of the study.

For peer review only - http://bmjopen.bmj.com/site/about/guidelines.xhtml

Results

Systematic search

Results of the systematic search are displayed in Figure 1. After removal of duplicates, the search returned 634 articles, of which 35 were retained after screening based on title/abstract. Following full text screening, 15 articles were retained for data extraction.

Textual synthesis

Description of the selected articles

Descriptive information about the selected articles is displayed in Table 3. Five of the selected articles were from the UK and the others were from the US (n = 7) or Australia (n = 3). A total of six clinical cases were reviewed across the 15 articles (see Table 3), as seven (47%) of the articles reported the same case (Case A, a well-publicised case of a 26 year old woman who died in the UK). Two of the clinical cases presented fictional scenarios.

Examination of clinical cases discussed in the selected articles

Specific information about clinical cases and decision-making is summarised and charted in Table 4. We only included examination of the factual cases (n = 6) in this part of the analysis, because we were interested in the types of real-world cases and decisions made, rather than an examination of a hypothetical scenario.

Patients discussed in the clinical cases varied in age, ranging from 26-86 years old. All patients were noted as having a diagnosis of depression, some were reported as also having diagnoses of Post traumatic stress disorder and personality disorders. The suicide methods used in the cases included self-poisoning (n = 3), gunshot incidents (n =2) and hanging (n =1). All patients were found by other people, except one patient who called an ambulance because they did not want to die alone. Four of the patients were reported to have died; the outcome in one case was not specified.

For peer review only

Treatment was provided in only one of the clinical scenarios. In this case, the patient was a psychiatric inpatient and the advance decision was considered part of the suicide attempt, so the patient's treatment refusal specified in the advance decision document was not adhered to.

The rationale for non-treatment in the clinical cases where the patient died varied and was summarised into the following three reasons:

- Advance decision was followed as a *legally-binding document* after checks showed the information was clear and specific, patient was informed of treatment options, had mental capacity at the time of writing and family were in agreement with the decision for non-treatment (n = 1).
- Physical injuries were severe resulting in *poor prognosis* for the patient and the treatment refusal in the advance decision was used as evidence that the patient would not wish to survive with a life-threatening or severely disabling condition. Where possible, families were also consulted (n = 2).
- *Verbal treatment refusal* was used as the basis for the treatment decision, rather than the advance decision, because the patient was conscious and had mental capacity. Consultation with family was not reported in this case. (n =1).

The decision-making process was reported to take considerable time and legal and/or ethical consultation took place in all the reported clinical cases. Differences in decision-making between emergency department clinicians and psychiatric consultants were reported in some of the clinical cases. In those cases, emergency department clinicians gave more weight to the advance decision, in contrast to psychiatrists who viewed suicide as a consequence of a distressed state and expressed a preference to treat the patient. Where conflict arose this was resolved through consultation with the hospital legal team and/or ethics committee.

For peer review only

Table 3. Description of selected studies

Article ID	Author	Date	Country	Perspective#	Fictional/ Factual case	Case reported*
1	Bryne	2002	Australia	Nursing	Fictional	--
2	Callaghan & Ryan	2011	Australia	Bioethics	Factual	A
3	Crippen et al	2001	US, Philadelphia, New York, New Zealand	Emergency & Acute medicine/ Bioethics	Factual	B
4	Cook et al	2010	US, Illinois	Psychiatry	Factual	C
5	Dresser	2010	US, New York	Legal	Factual	A
6	David et al	2010	UK	Psychiatry	Factual	A
7	Frank	2013	US, Colorado	Legal	Factual	D
8	Kapur et al	2010	UK	Psychiatry	Factual	E
9	Mitchell	2011	US, San Diego	Ethical	Fictional	--
10	Muzaffer	2011	UK	Psychiatry	Factual	A
11	Richardson	2013	UK	Legal	Factual	A
12	Ryan & Callaghan	2010	Australia	Psychiatry	Factual	A
13	Sontheimer	2008	US, Springfield	Bioethics	Factual	E
14	Szawarski	2013	UK	Bioethics	Factual	A
15	Volpe et al	2012	US, New York	Bioethics	Factual	F

Note: *For specific details about each case see Table 4, note fictional cases have not been given a case report ID
#where the perspective is not clearly stated this has been derived from the author(s) background and professional experience

For peer review only

Table 4. Description of clinical cases discussed in selected studies

Case ID	Citing Article(s)	Age	Mental health conditions	Nature of SA	Resulting Injuries/ illness	Hospital admittance	Nature of the AD	When written?	Patient conscious?	Decision Making process	Rationale for decision	Outcome
A	2,5,6,10, 11,12,14	26	Depression generalised anxiety disorder, PTSD, BPD	Self-poisoning (anti-freeze)	Not stated	Presented herself at hospital	Letter	3 days prior	Yes	Medical staff discussed the patient's mental capacity and sought legal advice.	The patient's wishes were clear in the letter but the patient was conscious, judged to have capacity and refusing treatment.	Death
B	3	46	Severe depression	Gunshot to face	Pain and severe facial injury	Gunshot reported by neighbours	Suicide note	not stated	Yes (not coherent)	The attending physicians thought life-support should be removed as the patient's "will" was clear and authoritative. The psychiatrist thought suicide was pathological and the condition was treatable so the patient should be treated. Clinicians consulted widely and sought legal advice	The suicide note was accepted as a living will. The patient had a desire to die due to psychological pain. The suicide attempt left the patient in a severely disabled state.	Death
C	4	57	Depression generalised anxiety disorder, PTSD, BPD	Self-poisoning (opiates)	Respiratory distress	Psychiatric inpatient	DNR	Prior to inpatient admittance	Not stated	There was conflict between clinicians; the psychiatrist argued that the DNR should not be followed because it was a suicide attempt. The legal/ethics committee was consulted who supported continued treatment.	DNR considered an effort to prepare for a suicide attempt and should not be honoured.	Survived and regretted the suicide attempt.
D	7	35	Depression and drug abuse	Hanging	Brain injury	Found by family	AD	Not stated	No	There were concerns that adherence to the AD would result in the patient's death. Clinicians sought legal advice.	The patient had poor prognosis and the family gave consent for clinicians to stop treatment.	Death
E	8,13	52	Depression generalised anxiety disorder, PTSD, BPD	Self-poisoning (insulin)	Coma	Found at home	AD	2 years prior	No	The AD mentioned no treatment for a terminal condition. The patient was not in a terminal condition and there were concerns that injury was the result of a suicide attempt and whether the AD should be adhered to in a suicidal context. Approached family and held an ethics committee consultation.	The patient's wishes were judged to be clear, the patient was considered to be informed about treatment options and had mental capacity at the time of writing the AD and the family were in agreement.	Death
F	15	86	Not stated	Gunshot to chest	Damage to pancreas and colon	Not stated	AD	Not stated	Yes (not always coherent)	Medical team argue that the nature in which the physical condition was caused (i.e. suicidal behaviour) should impact on treatment	Not stated	Not stated

Note: *for details about articles see Table 3, SA = suicide attempt, AD = advance directive, PTSD = post-traumatic stress disorder, BPD = borderline personality disorder

For peer review only - http://bmjopen.bmj.com/site/about/guidelines.xhtml

Thematic analysis

Five themes arose from the thematic analysis and are presented with their corresponding sub-themes and vote-counts in Table 5. We included accounts of fictional cases in the thematic analysis because here we were interested in opinions, views and perspectives of authors.

For peer review only

Table 6. Themes from the selected articles

Theme	Sub-themes	Theme Descriptor	Perspectives	Source(s)	Count %
Tension between patient autonomy and protecting a vulnerable person	*Professional dilemma: promoting patient autonomy vs. providing appropriate care*<br><br>*Societal expectation to protect vulnerable person and prevent suicide*	Tension between acting in accordance with patients' wishes for their medical treatment while promoting their best interests presented clinicians with a professional ethical dilemma. Clinicians also had a personal ethical dilemma, as there is societal pressure to protect vulnerable people and prevent suicide.	Psychiatry, Bioethics, Legal	2, 4, 7, 10, 12	5 (33%)
Appropriateness of advance decisions for suicidal behaviour	*Mental health symptoms and suicidal ideation fluctuate*<br><br>*Advance decisions for mental and physical health conditions – are they the same?*	There were questions about whether an advance decision "fits" in relation to suicide without an existing physical illness because mental state, mental health and suicide ideation fluctuate. Such scenarios are different from decisions made about treatment for a chronic or terminal physical condition.	Medical, Psychiatry, Bioethics, Legal	1, 3, 4, 5, 6, 7, 8, 10, 12, 13, 14, 15	12 (80%)
Uncertainty about the application of legislation	*Confusion and anxiety about litigation*<br><br>*Advance decisions are about more than a simple assessment of capacity*	Legislation around advance decisions was seen as confusing and there was anxiety about ligation. It was noted that mental capacity legislation overlapped with mental health legislation and policy. There were concerns that relying on a capacity decision was not sufficient and the authenticity of the advance decision needed to be considered	Medical, Psychiatry, Bioethics, Legal	1, 2, 3, 4, 5, 8, 9, 10, 11, 12, 14	11 (73%)
The length of time needed to consider all the evidence vs. rapid decision-making for treatment	*Need to fully consider the totality of evidence*<br><br>*Increased gravity of the clinical decision*	Clinical decisions were considered to be complex, involving an assessment of mental capacity, verification of the advance decision, and consideration of contextual factors. Therefore sufficient time was needed in which to consider all of the evidence.	Medical, Psychiatry, Bioethics Legal	1, 5, 8, 9, 10	5 (33%)
Importance of seeking support and sharing the decision	*Drawing up an advance decision as a collaborative process*<br><br>*Shared decision making*	Sharing the decision-making and seeking support, both at the time of writing the advance decision and when treating the patient, was viewed as important.	Medical, Psychiatry, Bioethics, Legal	1, 4, 5, 7, 9, 10, 11, 13, 14	9 (60%)

For peer review only

For peer review only - http://bmjopen.bmj.com/site/about/guidelines.xhtml

Themes

1) Tension between patient autonomy and protecting a vulnerable person

Professional dilemma: promoting patient autonomy vs. providing appropriate care

The management of an advance decision in the context of suicidal behaviour was particularly challenging because it went against healthcare professionals' training to preserve life (i.e. adherence to the advance decision could result in the death of the patient while they could recover if they received treatment for their physical condition). This presented clinicians with a dilemma between promoting patients' autonomy by observing their wishes stated in the advance decision and by providing care that was considered in their best interests (e.g. promoting life).

Societal expectation to protect vulnerable person and prevent suicide

Authors also raised the issue that clinicians not only had a professional interest in protecting a vulnerable person, but there was also a societal expectation that suicide should be prevented. The challenge to clinicians was highlighted by an acknowledgement from some authors that adherence to the advance decision in this context was emotive and would feel like assisting suicide.

2) Appropriateness of advance decisions for suicidal behaviour

Mental health symptoms and suicidal ideation fluctuate

Concerns were expressed about whether an advance decision should apply in the context of suicidal behaviour because of patients' distressed state, the potential for suicidal ideation to fluctuate and for treatment preferences to change in the future.

For peer review only - http://bmjopen.bmj.com/site/about/guidelines.xhtml

Authors from a psychiatric perspective, in particular, viewed suicidal behaviour as a symptom of a mental health condition that was potentially treatable with psychiatric care. They also expressed concerns about the capacity of a distressed suicidal person to fully comprehend their decision and consider all treatment options available to them. Therefore, it was suggested by some authors that a higher level of mental capacity may be required at the time of writing the advance decision for clinicians to be confident in following it. However, other authors argued that the advance decision should be considered as part of the suicide attempt and as evidence of distressed thinking, rather than independently of the attempt, and the treatment refusal in the advance decision document should not be adhered to.

Advance decisions for mental and physical health conditions – are they the same?

The difference between an advance decision for suicidal behaviour and for a physical condition was highlighted across the selected papers. Authors from a legal perspective highlighted that the primary aim of an advance decision relating to a suicide attempt is to end life, whereas an advance decision for a chronic or terminal illness is often concerned with managing pain and avoiding prolonged suffering.

There was also debate about the extent to which mental suffering legitimised suicide. Authors from an ethical perspective argued that, typically, healthcare services may be more sympathetic to "end of life" decisions relating to terminal physical health conditions than mental health conditions, thus mental health patients do not receive the same palliative care options as patients without mental health diagnoses. There was some discussion that it should not be assumed that psychiatric pain is more tolerable than physical pain and that both should be considered as having a similar influence on the patient.

3) Uncertainty about the application of legislation

Confusion and anxiety about litigation

For peer review only - http://bmjopen.bmj.com/site/about/guidelines.xhtml

Authors from general medical and psychiatry perspectives expressed confusion about legislation and anxiety about litigation, with one stating that the advance decision document needed to be *'watertight'* to be considered evidence of the patient's. Authors recommended that clear hospital policies be developed for advance decisions in this particular context to overcome the confusion and anxiety about ligation.

Authors from the UK and Australia highlighted the difficulties in implementing both mental health and mental capacity legislation when managing advance decisions relating to suicidal behaviour. Clinicians needed to consider whether someone who had attempted suicide was suffering with a mental health condition, for which they should be treated against their will. They also needed to judge whether the person had the capacity to make a decision about their treatment and, if so, that the advance decision could apply following verification checks. Some suggested that application of each legislation model (i.e. mental health or mental capacity), in isolation of the other, could result in different outcomes for the patient. Some authors suggested that the difficulty with balancing mental capacity legislation and mental health legislation could be resolved by developing a single legislation that combines both.

Advance decisions are about more than a simple assessment of capacity

A reliance on judging a person's capacity to make a decision in the context of suicidal behaviour was discussed in detail. While this is an important part of some legislation, particularly in the UK, it was suggested that an assessment of capacity should be supplemented with a judgment of the authenticity and durability of the patient's decision (i.e. if the decision had been consistent over time). Authors from a psychiatric perspective, in particular, suggested that advance decisions should be regularly reviewed to ensure that they were up-to-date and continued to reflect the patient's desires and preferences.

For peer review only

4) The length of time needed to consider all the evidence vs. rapid decision-making for treatment

Need to fully consider the totality of evidence

Some authors suggested that the increased length of time taken in this particular context arose from the need to consider contextual factors for the suicidal behaviour, the patient's mental health background and the reason for their decision, alongside the usual validation checks and judgment as to presence of mental capacity at the time of making the advance decision. It was also argued that clinicians should take into account wider factors that may have not been present when the person first wrote the advance decision, such as technological advances offering new treatment options that may influence the patient's decision.

However, authors highlighted difficulties with gaining access to such evidence, particularly in emergency situations, further adding to the time taken to make a decision. It was noted that advance decisions were often too specific or too general, resulting in ambiguity as to the best course of action for the patient and time consuming investigation. Some authors highlighted that advance decisions were not useful in emergency settings when rapid decision-making was required. Advance decisions may be more appropriate for patients to express refusals of on-going psychiatric treatment (e.g. Electroconvulsive therapy).

Increased gravity of the clinical decision

Authors argued that the gravity of the clinical decision was increased in this context because the patient could die if the advance decision was adhered to when recovery from mental ill health may be possible. Authors suggested that validation checks in this context may need to be more thorough and authors from a legal perspective argued that, because of the increased gravity of the clinical decision, physicians should seek a consensus about clinical management, whilst providing life-sustaining treatment, creating a time-consuming situation.

For peer review only - http://bmjopen.bmj.com/site/about/guidelines.xhtml

5) Importance of seeking support and sharing the decision

Drawing up an advance decision as a collaborative process

Some authors argued that when writing an advance decision, patients should be supported by a healthcare professional to consider all possible treatment options. It was suggested that evidence of mental capacity at the time of writing the advance decision should be provided (e.g. verified and signed by the healthcare professional) which could help with clinical decision-making at a later stage. Authors from all the perspectives stressed the importance of also consulting with a physician at the time of writing the advance decision to ensure that it is both specific and general enough to be helpful and informative in a given medical scenario.

Shared decision making

All authors discussed the need for multi-agency decision-making in relation to the management of advance decisions in the context of suicidal behaviour. Suggestions included that clinicians should consult widely, make use of psychiatric expertise, review the patient's psychiatric history and background and seek legal and/or ethical consultation when considering treatment decisions.

Discussion

Summary of the findings

There were inconsistent views on practice and rationales for the management of advance decisions. Conflict between clinicians and uncertainty about decision-making were reported. Despite the legislation relating to advance decisions, some questioned whether an advance decision in this particular context was legally binding. The appropriateness of advance decisions with suicidal behaviour was questioned because suicide ideation fluctuates and outcomes for treatment refusal in this context may be different to those for a terminal

For peer review only

physical health condition (i.e. the patient could die when there is potential for recovery in the future). There was only one case reported where treatment was given and the patient survived and in that case the patient later regretted the suicide attempt. Management of the advance decision was difficult both emotionally and ethically for some clinicians because it challenged their professional training and their desire to protect vulnerable patients from suicide.

The competing pressures of respecting individuals' rights to autonomy while protecting them from the effects of mental disorder found in the current study are a commonly reported dilemma (Allen, 2013). There is evidence from the present study that support for the right to autonomy may be more dominant in clinicians from emergency medicine disciplines, with those from a psychiatric background prioritising prevention of suicide. A 'middle ground' between these views may help to provide guidance for clinicians. For example, in English law, courts have acknowledged that while some suicidal individuals may have capacity, the overwhelming likelihood is that capacity is impaired to at least some degree (Allen, 2013). Therefore a higher degree of certainty should be required when assessing capacity with suicidal behaviour and clinicians should err on the side of caution (Kapur et al., 2010b). Another potential resolution to this dilemma, particularly in emergency scenarios, may be to provide 'temporary intervention' to allow time for individuals to be assessed and treatment options to be discussed (Allen, 2013).

Recommendations for practice

Decisions made about advance decisions in the context of suicidal behaviour should be made in full consultation with psychiatric teams and with relevant legal and/or ethical advisers. The results also highlight the importance of allocating sufficient time to consider contextual evidence relating to the suicidal behaviour, the authenticity of the treatment decision and verification of the documentation/decision. Given the gravity and emotive

For peer review only

nature of a decision in this context, emergency healthcare workers may need increased support and supervision for such incidents.

Findings indicate that it may be helpful, in this particular context, for an advance decision to be written in consultation with a professional healthcare worker. This practice would also ensure that the patient is supported to consider all treatment options, that the advance decision is specific and detailed enough to be useful in an emergency situation and that patients' capacity at the time of writing the advance decision can be assessed and verified. The advance decision should be regularly reviewed and updated to ensure that it reflects the patient's current treatment decisions.

Strengths and Limitations

A strength of this review is that a broad range of articles from different disciplines were included, thus increasing the generalisability of results. However, there were some potential biases in the literature. First, there was a paucity of evidence: only six clinical cases were reported across the selected articles. There was also a risk of bias from the studies themselves, given that they were reviews of single clinical cases. Second, the articles were focussed on the US, UK and Australia, so may have resulted in bias relating to the specific legislation/ethics of those countries. It will be important for future research to compare findings internationally across a wider range of countries (Blank, 2011; Mishara & Weisstub, 2016, van Wijmen et al., 2010). Third, as with any syntheses of qualitative data there was potential for bias to be introduced by the research team at the stages of study identification, data extraction and synthesis. This was minimised in the current study by having two researchers carry out these tasks independently and cross-check the findings.

For peer review only

Future directions

Empirical studies, such as interviews and focus groups with clinicians and patients and/or a national clinical survey are important future priorities. Research examining the prevalence of advance decisions relating to suicidal behaviour could shed light on the frequency of such presentations. Suitable platforms for storing advance decisions could also be explored. For example, some have suggested a web application ('app') could better reflect the dynamic nature of treatment refusal (Huxtable, 2015) and make updating and reassessment easier.

Conclusion

Current literature on the management of advance decisions and suicidal behaviour centres on detailed accounts of clinical cases and demonstrates variability in practice and the rationale behind clinical decisions. Challenges in managing advance decisions specific to suicidal behaviour were evident and there was some debate about whether advance decisions in the context of suicidal behaviour were appropriate in their current form. Taking time to consider all the evidence when making a decision, consulting fully with mental health clinicians and seeking legal and/or ethical advice may help with some of these challenges. The support of a relevant healthcare professional at the time of writing the advance decision may also be useful.

For peer review only

For peer review only - http://bmjopen.bmj.com/site/about/guidelines.xhtml

Selected journal articles:

1. Byrne, M. The use of advance directives. The use of advance directives. 2002. Nursing Monograph, 13-16.

2. Callaghan, S. & Ryan, C. J. Refusing medical treatment after attempted suicide: Rethinking capacity and coercive treatment in light of the Kerrie Wooltorton case. Journal of Law and Medicine. 2011. 18 (4) 811-819

3. Chalfin DB, Crippen D, Franklin C, Kelly DF, Kilcullen JK, Streat S, et al. 'Round-table' ethical debate: Is a suicide note an authoritative 'living will'? Critical Care. 2001;5(3):115-24.

4. Cook R, Pan P, Silverman R, Soltys SM. Do-not-resuscitate orders in suicidal patients: Clinical, ethical, and legal dilemmas. Psychosomatics: Journal of Consultation and Liaison Psychiatry. 2010;51(4):277-82.

5. David A, Hotpur, M, Mora, P, Owen, G, Szmukler, G, Richardson, G. Mentally disordered or lacking capacity? Lessons for managing serious deliberate self-harm: Advance directives and suicidal behaviour. British Medical Journal. 2010;341, 587-589.

6. Dresser, R. Suicide attempts and treatment refusals. 2010. Hastings Centre Report, 40(30), 10-11.

7. Frank, C. How to reconcile advance care directives with attempted suicide. The Colorado Lawyer. 2013;42(7):97-101.

8. Kapur, N, Clements, C, Bateman, N, Foex, B, Mackway-Jones, K, Huxtable, R, Gunnell, D, Hawton, K. Advance directives and suicidal behaviour. British Medical Journal. 2010; 341, 590-91.

9. Mitchell, M. An analysis of common arguments against advance directives. 2011. Nursing Ethics, 19 (2) 245-51.

10. Muzaffar S. 'To treat or not to treat'. Kerrie Wooltorton, lessons to learn. Emergency Medicine Journal. 2011;28(9):741-4.

11. Richardson, G. Mental capacity in the shadow of suicide: What can the law do? International Journal of Law in Context, 2013, 9 (1), 87-105.

12. Ryan, C., J. & Callaghan, S. C. Legal and ethical aspects of refusing medical treatment after a suicide attempt: the Wooltorton case in the Australian context. Clinical Practice. 2010: 193 (4) 239-243.

For peer review only

13. Sontheimer D. Suicide by advance directive? Journal of Medical Ethics. 2008;34(9):e4-e 1p.

14. Szawarski P. Classic cases revisited: The suicide of Kerrie Wooltorton, The Intensive Care. 14: e4

15. Volpe, R. L., Levi, B. H., Blackall, G. F., & Green, M. J. 2012. Exploring the limits of autonomy. Hastings Centre Report, 16-18.

For peer review only

References

Allen N. The right to life in a suicidal state. International journal of law and psychiatry. 2013 Sep 1;36(5-6):350-7. doi: 10.1016/j.ijlp.2013.09.002

Blank RH. End-of-life decision making across cultures. The Journal of Law, Medicine & Ethics. 2011 Jun 1;39(2):201-14. doi: 10.1111/j.1748-720X.2011.00589.x

Brinkman-Stoppelenburg A, Rietjens JA, van der Heide A. The effects of advance care planning on end-of-life care: a systematic review. Palliative medicine. 2014 Sep;28(8):1000-25. doi: 10.1177/0269216314526272

Campbell LA, Kisely SR. Advance treatment directives for people with severe mental illness. The Cochrane Library. 2009 Jan 1. doi: 10.1002/14651858

Carroll R, Metcalfe C, Gunnell D. Hospital presenting self-harm and risk of fatal and non-fatal repetition: systematic review and meta-analysis. PLoS One. 2014 Feb 28;9(2):e89944. doi: 10.1371/journal.pone.0089944

Centre for reviews and dissemination, Undertaking systematic reviews of research on effectiveness, CRD's guidance for carrying out or commissioning reviews, 2009, York, University of York, 3

Dresser R. Suicide attempts and treatment refusals. Hastings Center Report. 2010;40(3):10-1. doi: 10.1353/hcr.0.0269

Department of Health. Mental Capacity Act. London: Stationery Office, 2005.

Frank C. How to Reconcile Advance Care Directives With Attempted Suicide. Colo. Law.

For peer review only

2013 Jul:97-8.

Hawton K, Bergen H, Casey D, Simkin S, Palmer B, Cooper J, Kapur N, Horrocks J, House A, Lilley R, Noble R. Self-harm in England: a tale of three cities. Social psychiatry and psychiatric epidemiology. 2007 Jul 1;42(7):513-21. doi: 10.1007/s00127-007-0199-7

Houben CH, Spruit MA, Groenen MT, Wouters EF, Janssen DJ. Efficacy of advance care planning: a systematic review and meta-analysis. Journal of the American Medical Directors Association. 2014 Jul 31;15(7):477-89. doi: 10.1016/j.jamda.2014.01.008

Huxtable R. Advance decisions: worth the paper they are (not) written on?. End of Life Journal. 2015 Sep 1;5(1):e000002.doi:10.1136/eoljnl-2015-000002

Kapur N, Clements C, Bateman N, Foëx B, Mackway-Jones K, Hawton K, Gunnell D. Self-poisoning suicide deaths in England: could improved medical management contribute to suicide prevention?. QJM: An International Journal of Medicine. 2010 Aug 4;103(10):765-75. doi: 10.1093/qjmed/hcq128

Kapur N, Clements C, Bateman N, Foëx B, Mackway-Jones K, Huxtable R, Gunnell D, Hawton K. Advance directives and suicidal behaviour. BMJ: British Medical Journal (Online). 2010 Sep 7;341. doi: 0.1136/bmj.c4557

Murad MH, Sultan S, Haffar S, et al. Methodological quality and synthesis of case series and case reports BMJ Evidence-Based Medicine Published Online First: 02 February 2018. doi: 10.1136/bmjebm-2017-110853

Mishara BL, Weisstub DN. The legal status of suicide: A global review. International journal of law and psychiatry. 2016 Jan 1;44:54-74. doi: 10.1016/j.ijlp.2015.08.032

For peer review only

Moher D, Shamseer L, Clarke M, Ghersi D, Liberati A, Petticrew M, Shekelle P, Stewart LA. Preferred reporting items for systematic review and meta-analysis protocols (PRISMA-P) 2015 statement. Systematic reviews. 2015 Dec;4(1):1. doi: 10.1186/2046-4053-4-1

Spencer L, Ritchie J. Qualitative data analysis for applied policy research. In Analyzing qualitative data 2002 Sep 9 (pp. 187-208). Routledge.

Ryan R; Cochrane Consumers and Communication Review Group. Cochrane Consumers and Communication Review Group: data synthesis and analysis'. http://cccrg.cochrane.org, June 2013 (accessed 25th April 2018).

Shamseer L, Moher D, Clarke M, Ghersi D, Liberati A, Petticrew M, Shekelle P, Stewart LA. Preferred reporting items for systematic review and meta-analysis protocols (PRISMA-P) 2015: elaboration and explanation. Bmj. 2015 Jan 2;349:g7647. doi. 10.1136/BMJ.g7647.

Swanson JW, Swartz MS, Elbogen EB, Van Dorn RA, Ferron J, Wagner HR, McCauley BJ, Kim M. Facilitated psychiatric advance directives: a randomized trial of an intervention to foster advance treatment planning among persons with severe mental illness. American Journal of Psychiatry. 2006 Nov;163(11):1943-51. doi: 10.1176/ajp.2006.163.11.1943

Van Wijmen MP, Rurup ML, Pasman HR, Kaspers PJ, ONWUTEAKA-PHILIPSEN BD. Advance directives in the Netherlands: An empirical contribution to the exploration of a cross-cultural perspective on advance directives. Bioethics. 2010 Mar 1;24(3):118-26. doi: 10.1111/j.1467-8519.2009.01788.x

For peer review only

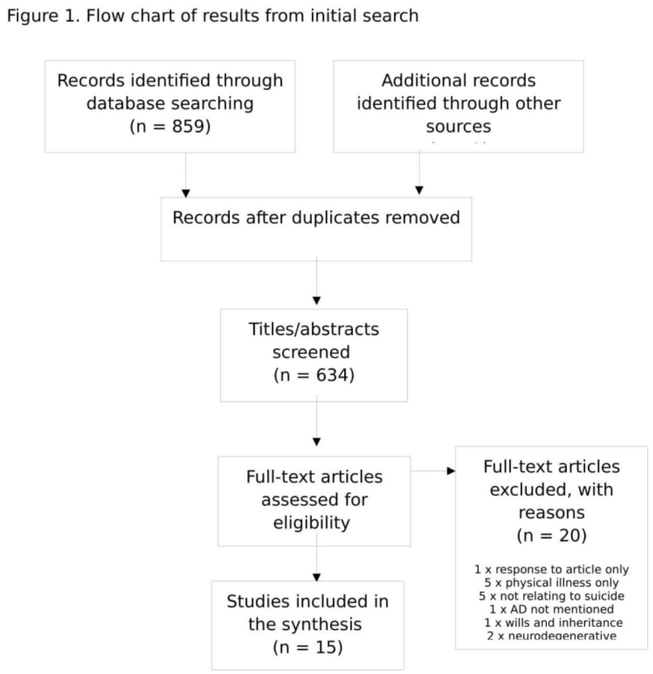

Figure 1

139x198mm (300 x 300 DPI)

PRISMA 2009 Checklist

Section/topic	#	Checklist item	Reported on page #
**TITLE**			
Title	1	Identify the report as a systematic review, meta-analysis, or both.	1
**ABSTRACT**			
Structured summary	2	Provide a structured summary including, as applicable: background; objectives; data sources; study eligibility criteria, participants, and interventions; study appraisal and synthesis methods; results; limitations; conclusions and implications of key findings; systematic review registration number.	4
**INTRODUCTION**			
Rationale	3	Describe the rationale for the review in the context of what is already known.	7
Objectives	4	Provide an explicit statement of questions being addressed with reference to participants, interventions, comparisons, outcomes, and study design (PICOS).	7
**METHODS**			
Protocol and registration	5	Indicate if a review protocol exists, if and where it can be accessed (e.g., Web address), and, if available, provide registration information including registration number.	No protocol
Eligibility criteria	6	Specify study characteristics (e.g., PICOS, length of follow-up) and report characteristics (e.g., years considered, language, publication status) used as criteria for eligibility, giving rationale.	8 (Table 1 and 2)
Information sources	7	Describe all information sources (e.g., databases with dates of coverage, contact with study authors to identify additional studies) in the search and date last searched.	8
Search	8	Present full electronic search strategy for at least one database, including any limits used, such that it could be repeated.	8 (Table 1 and 2)
Study selection	9	State the process for selecting studies (i.e., screening, eligibility, included in systematic review, and, if applicable, included in the meta-analysis).	8 (Table 1 and 2)
Data collection process	10	Describe method of data extraction from reports (e.g., piloted forms, independently, in duplicate) and any processes for obtaining and confirming data from investigators.	9
Data items	11	List and define all variables for which data were sought (e.g., PICOS, funding sources) and any assumptions and simplifications made.	9
Risk of bias in individual studies	12	Describe methods used for assessing risk of bias of individual studies (including specification of whether this was done at the study or outcome level), and how this information is to be used in any data synthesis.	10
Summary measures	13	State the principal summary measures (e.g., risk ratio, difference in means).	n/a

For peer review only

PRISMA 2009 Checklist

Synthesis of results	14	Describe the methods of handling data and combining results of studies, if done, including measures of consistency (e.g., I^2) for each meta-analysis.	9

Section/topic	#	Checklist item	Reported on page #
Risk of bias across studies	15	Specify any assessment of risk of bias that may affect the cumulative evidence (e.g., publication bias, selective reporting within studies).	10
Additional analyses	16	Describe methods of additional analyses (e.g., sensitivity or subgroup analyses, meta-regression), if done, indicating which were pre-specified.	9
**RESULTS**			
Study selection	17	Give numbers of studies screened, assessed for eligibility, and included in the review, with reasons for exclusions at each stage, ideally with a flow diagram.	10 (Figure 1)
Study characteristics	18	For each study, present characteristics for which data were extracted (e.g., study size, PICOS, follow-up period) and provide the citations.	10-12, 21 (Table 3)
Risk of bias within studies	19	Present data on risk of bias of each study and, if available, any outcome level assessment (see item 12).	n/a
Results of individual studies	20	For all outcomes considered (benefits or harms), present, for each study: (a) simple summary data for each intervention group (b) effect estimates and confidence intervals, ideally with a forest plot.	Textual analysis 10-12
Synthesis of results	21	Present results of each meta-analysis done, including confidence intervals and measures of consistency.	Synthesis of Qual results 12-17
Risk of bias across studies	22	Present results of any assessment of risk of bias across studies (see Item 15).	Noted in discussion p19
Additional analysis	23	Give results of additional analyses, if done (e.g., sensitivity or subgroup analyses, meta-regression [see Item 16]).	n/a
**DISCUSSION**			
Summary of evidence	24	Summarize the main findings including the strength of evidence for each main outcome; consider their relevance to key groups (e.g., healthcare providers, users, and policy makers).	17-18
Limitations	25	Discuss limitations at study and outcome level (e.g., risk of bias), and at review-level (e.g., incomplete retrieval of identified research, reporting bias).	19

For peer review only

PRISMA 2009 Checklist

Conclusions	26	Provide a general interpretation of the results in the context of other evidence, and implications for future research.	20
**FUNDING**			
Funding	27	Describe sources of funding for the systematic review and other support (e.g., supply of data); role of funders for the systematic review.	2

From: Moher D, Liberati A, Tetzlaff J, Altman DG, The PRISMA Group (2009). Preferred Reporting Items for Systematic Reviews and Meta-Analyses: The PRISMA Statement. PLoS Med 6(7): e1000097. doi:10.1371/journal.pmed1000097

For more information, visit: **www.prisma-statement.org**.

For peer review only

BMJ Open

The management of patients with an advance decision and suicidal behaviour: A systematic review

Journal:	*BMJ Open*
Manuscript ID	bmjopen-2018-023978.R1
Article Type:	Research
Date Submitted by the Author:	04-Sep-2018
Complete List of Authors:	Nowland (Harris), Rebecca; University of Manchester, Centre for Health and safety<br>Steeg, Sarah; University of Manchester, Psychiatry<br>Quinlivan, Leah; University of Manchester , Brain, Behaviour and Mental Health<br>Cooper, Jayne; University of Manchester,<br>Huxtable, Richard; University of Bristol, School of Social and Community Medicine<br>Hawton, Keith; Centre for Suicide Research, Oxford University, Psychiatry<br>Gunnell, DJ; University of Bristol, Social Medicine<br>Allen, Neil; University of Manchester, School of Law<br>Mackway-Jones, Kevin; Manchester Royal Infirmary, Emergency Department<br>Kapur, Navneet; University of Manchester, Centre for suicide prevention
**Primary Subject Heading**:	Mental health
Secondary Subject Heading:	Emergency medicine, General practice / Family practice, Medical management
Keywords:	Suicide & self-harm < PSYCHIATRY, suicidal behaviour, advance decisions, advance directives, living wills

SCHOLARONE™
Manuscripts

Title: The management of patients with an advance decision and suicidal behaviour: A systematic review

Author list: Rebecca Nowland, PhD[1]; Sarah Steeg, PhD[1]; Leah Quinlivan, PhD[1,2]; Jayne Cooper, PhD[1], Prof Richard Huxtable[3]; Prof Keith Hawton, FMedSc[4]; Prof David Gunnell DSc[5], Neil Allen (Barrister)[6], Prof Kevin Mackway-Jones[7], Prof Nav Kapur, FRPsych[1,2, 8]

1. Centre for Suicide Prevention, Manchester Academic Health Science Centre, University of Manchester, England
2. NIHR Greater Manchester Patient Safety Translational Research Centre, England
3. Centre for Ethics in Medicine, University of Bristol, England
4. Centre for Suicide Research, University Department of Psychiatry, Warneford hospital, Oxford, England
5. Department of Population Health Sciences, University of Bristol, England
6. School of Law, University of Manchester, England
7. Emergency Department, Manchester Royal Infirmary, Manchester University NHS Foundation Trust, Manchester, England
8. Greater Manchester Mental Health NHS Foundation Trust, Manchester, England.

Corresponding author: Dr Rebecca Nowland

Address: School of Nursing, Brook Building, University of Central Lancashire, PR1 2HE

Telephone: +44 (0)161 275 0727

Email: RNowland@uclan.ac.uk

Word count = 5853 (excluding abstract and references)

For peer review only

Authorship and contribution

All authors made substantial contributions to the study. RN and LQ conducted the initial scoping search. RN designed the review and data extraction/analysis with input from NK and SS. RN and SS screened and reviewed the articles and performed data extraction/analysis, interpreted the results and wrote the first draft. NK, JC, RH, KH, DG, NA, and KMJ reviewed the initial draft, contributed to subsequent drafts and approved the final version. All authors take responsibility for the integrity of the data analysis. NK is the guarantor of the study.

Declaration of Interest

DG, KH, and NK are members of the Department of Health's (England) National Suicide Prevention Advisory Group. NK chaired the NICE guideline development group for the longer term management of self-harm and the NICE Topic Expert Group (which developed the quality standards for self-harm services). He is currently chair of the updated NICE guideline for Depression. KH and DG are NIHR Senior Investigators. KH is also supported by the Oxford Health NHS Foundation Trust and NK by the Greater Manchester Mental Health NHS Foundation Trust.

Funding

This paper presents independent research funded by the National Institute of Health Research (NIHR) under its Programme Grants for Applied Research Programme (Grant Reference Number RP-PG-0610-10026). The views expressed are those of the authors and not necessarily those of the NHS, the National Institute of Health Research or the Department of Health.

Data sharing statement

No additional data are available

Provenance and peer review

Not commissioned; externally peer reviewed

For peer review only

Acknowledgements

We would to thank Rosie Davies, Research Fellow in Patient and Public Involvement at the University of Bristol, for her input into the study.

For peer review only

ABSTRACT

Background: The use of advance care planning and advance decisions for psychiatric care is growing. However, there is limited guidance on clinical management when a patient presents with suicidal behaviour and an advance decision and no systematic reviews of the extant literature.

Objectives: To synthesise existing literature on the management of advance decisions and suicidal behaviour.

Design: A systematic search of 7 bibliographic databases was conducted to identify studies relating to advance decisions and suicidal behaviour. Studies on terminal illness or end of life care were excluded to focus on the use of advance decisions in the context of suicidal behaviour. A textual synthesis of data was conducted and themes were identified by using an adapted thematic framework analysis approach.

Results: Overall 634 articles were identified, of which 35 were retained for full text screening. Fifteen relevant articles were identified following screening. Those articles pertained to actual clinical cases or fictional scenarios. Clinical practice and rationale for management decisions varied. Five themes were identified: 1) tension between patient autonomy and protecting a vulnerable person, 2) appropriateness of advance decisions for suicidal behaviour, 3) uncertainty about the application of legislation, 4) the length of time needed to consider all the evidence vs. rapid decision-making for treatment, and 5) importance of seeking support and sharing decision-making.

Conclusions: Advance decisions present particular challenges for clinicians when associated with suicidal behaviour. Recommendations for practice and supervision for clinicians may help to reduce the variation in clinical practice.

Keywords: self-harm, suicidal behaviour, advance directives, advance decisions, living wills, Ulysses directives

For peer review only

Article Summary

Strengths and limitations of this study

- Timely systematic review considering the challenges relating to advance decisions in the context of suicidal behaviour
- Review involves journal articles from a variety of countries from a range of disciplines
- Paucity of evidence for this specific presentation of advance decision
- Evidence in this area is predominately from reviews of case studies, rather than empirical work

For peer review only

INTRODUCTION

An advance decision (sometimes known as an advance decision to refuse treatment (ADRT) or living will) is typically a written document that outlines a person's desire to refuse certain treatments, including life-saving treatment, when there is a potential for a person to lose the mental capacity to make treatment decisions in the future.[1] In order for an advance decision to be valid, the person must have mental capacity at the time of writing the document. Mental capacity is defined as the ability to make a decision and involves understanding and weighing information relating to a decision and alternative options and retaining that information long enough to make the decision.[1] The Mental Capacity Act in England and Wales refers to "advance decisions to refuse treatment (ADRT)," but more widely these documents are referred to as "advance directives" and/or "living wills". We use "advance decision" throughout in this paper to refer to written documents stating a refusal of treatment made in advance of medical treatment following an illness or injury.

There are important cross-national variations in legislation; in some countries, the use of advance decisions is not permitted (i.e. Turkey, Japan), while in others, advance decisions are legislated for (i.e. the UK and US). The UK, Australia and US have similar legal standards with some state-wide variation in the US and Australia,[2] with some states adopting the common law right to make an advance decision and others allowing the use of a surrogate or proxy decision maker (i.e. to make healthcare decisions on behalf of the patient). There is also considerable variation in practice between countries where advance decisions are permitted. For example, in Germany, advance decisions are recognised but require court approval in each case.[2]

Advance care planning for psychiatric care is becoming more common in a number of countries, including the UK, US and Australia[3, 4] and enables patients to state their preferences for the management of their mental health condition when they may temporarily lose their mental capacity. A person with a mental health condition may also make some decisions about particular treatment that they would not wish to have and may involve an advance decision to refuse particular treatments, i.e. electroconvulsive therapy. Advance care planning has been shown to have a number of healthcare benefits for mental

For peer review only

health patients in the UK and US, such as enhancing patient autonomy and engagement, promoting adherence to treatment plans (i.e. patients taking prescribed drugs), improving continuity of care with fewer psychiatric admissions, reducing the use of social workers' time and lower levels of violent acts.[3,4] In a recent survey of patients with bipolar, 21% had written statements about their healthcare, and of those, 10% involved an advance decision to refuse treatment.[5] This increasing use of advance care planning in mental health may result in an increasing use of advance decisions to refuse mental health care treatment, and concerns about clinical management of advance decisions following self-harm and/or suicide attempts have been made by healthcare professionals and legal and ethical consultants.[6-8] Existing literature, from a variety of academic and clinical perspectives, suggests there is little consistency in practice and there are specific challenges with advance decisions following self-harm or suicidal behaviour. Such scenarios raise questions about whether a person with a wish to end their life has the capacity to make a decision about refusal of treatment and/or if their capacity is affected by mental illness, and whether an advance decision is appropriate for medical treatment following suicidal behaviour.[8]

The management of self-harm and suicide attempts is a significant challenge for clinicians in the emergency services. Each year over 200,000 people present to emergency departments in England with self-harm,[9] with 16% of those presenting to hospital with a repeat self-harm episode within a year.[10] Treatment refusal following self-harm has been shown to be common. A prospective cohort study of mental capacity and self-harm in the ED found that around 40% of patients presenting to hospital with self-harm had the capacity to make a decision about their medical treatment and 30% of those intended to refuse life-saving treatment.[11] There are few studies that have examined numbers of advance decisions to refuse treatment in patients presenting with self-harm or suicidal behaviour, but in a recent study, three out of 121 fatal cases of self-poisoning in 2005, the patients had an advance decision.[12] Given that patient autonomy and advance care planning are encouraged in modern healthcare and are assuming greater prominence, it is likely that the number of people presenting to hospital with an advance decision following self-harm or a suicide attempt will grow.

For peer review only

Rationale

While reviews of literature relating to the management of advance decisions, both more broadly and specifically to relating to “end of life” care exist,[13, 14] there are currently no reviews on the management of advance decisions when a patient presents to hospital following self-harm or a suicidal attempt where the patient does not have a chronic or terminal physical illness. Despite the legislative context being similar for end of life care, the ethical considerations, emotional challenges and clinical decision-making may be different for treatment of a patient following suicidal behaviour without a chronic or terminal physical illness. A synthesis of this literature is important to examine similarities and differences and to establish the key findings, particularly as the management of advance decisions to refuse treatment of injuries and illnesses following self-harm or suicide attempts is challenging for clinicians[8] and there is a lack of consistency of practice. A review of the literature will be important to inform guidelines for the management of advance decisions following self-harm or suicidal behaviour.

Aim

To systematically review and synthesise literature on the treatment and clinical management of patients presenting to hospital with an advance decision to refuse treatment following suicidal behaviour without a chronic or terminal physical illness. The terminology for suicidal behaviour varies internationally. Some clinicians/researchers distinguish between suicide attempts and non-suicidal self-injury[15], while others prefer the broad term of self-harm to denote behaviours across the spectrum.[1, 9] We took an inclusive approach to ensure we captured relevant studies, so in this review we refer to “suicidal behaviour” as behaviours including all self-harming behaviour and suicide attempts. The review was conducted by researchers in the UK, but an examination of all the existing literature was conducted without language or country restrictions.

For peer review only - http://bmjopen.bmj.com/site/about/guidelines.xhtml

Method

The review was conducted in accordance with PRISMA guidelines[16, 17] and guidance for conducting narrative synthesis in healthcare.[18] There is no protocol for the review. We used the PRISMA checklist when writing our report.[16]

Search strategy and data sources

An initial scoping of the literature was conducted at inception of the study and the findings were used to inform the search strategy. Content experts and clinical practitioners on the research team assisted with compiling key words and/or phrases (see Table 1). In order to take an inclusive approach and enable inclusion of any papers that involved discussion of management of advance decisions following "suicidal behaviour" we included a variety of key search terms relating to non-accidental injury and suicidal attempts. An electronic search of six databases (EMBASE, MEDLINE, PSYCHINFO, Social Policy and Practice, CINAHL and Medline) was conducted, as well as a full electronic search on WestLaw (an online library of UK legal information) using the following search terms: *advance decisions, advance directives* AND *wills, suicide*. Full search strategy for each database is supplied as supplementary information (Supplementary Information 1). In addition, the reference sections of all included sources were consulted and authors' personal files were also searched to ensure that potentially eligible sources were not omitted. No study design, date or language restrictions were imposed.

Literature searches were conducted during the period April 2016 to July 2018. The specific inclusion and exclusion criteria are detailed in Table 2.

Study selection

Titles and abstracts were screened, with a random sample of 10% of the articles independently screened by another researcher. Additional information was sought where there were any disagreements, which were then resolved through discussion. An acceptable

For peer review only

concordance rate between the inclusion decisions was predefined as agreement on at least 90% of the articles, which was achieved for screening on title and abstract. Full text screening of the selected articles was conducted by two researchers independently, with full agreement being achieved at this stage.

Data extraction and analysis

A preliminary analysis of the data was conducted.[18] Studies were from a range of disciplines (i.e. general medical, psychiatry, ethical, legal) and involved reviews of clinical cases or fictional scenarios. It was deemed appropriate to conduct a narrative synthesis because this particular approach is useful when synthesising textual findings from diverse literatures.[18] Narrative synthesis was conducted in two phases: 1) a *textual synthesis*, and 2) an adapted *thematic framework analysis*.[19]

First, the *textual synthesis* of the data was conducted by extracting key factual information from each study (country of origin, perspective/discipline, factual or fictional case study) and details of the case studies (age of patient, mental health conditions, nature of suicidal behaviour, resulting injuries/illness, hospital admittance, type of advance decision, when advance decision was written, whether patient was conscious, decision-making processes, rationale for decision, outcome). The information was then summarised and tabulated to map the literature that cited the same clinical case. Information from cases only involving a factual case study (i.e. a real clinical case) was extracted because we were interested in information about actual clinical cases, decision-making process and rationale for decisions made. Thus, information was not extracted from reports that discussed a hypothetical scenario for the textual synthesis. Data extraction and summarisation was completed independently by two researchers using a pre-determined data extraction sheet.

Second, an adapted *thematic framework analysis* approach[19] was used to examine key themes discussed in the selected papers. This involved five stages: initial open coding, indexing, descriptive summaries, charting and tabulation and interpretation. *Initial open coding* generated three general categories representing the most discussed issues across

For peer review only

the selected articles: 1) key issues with an advance decision relating to suicidal behaviour, 2) challenges in clinical decision-making for advance decisions relating to suicidal behaviour, and 3) recommendations for practice. These three categories were used to index the data and as a framework to extract and summarise data. Extracted data was then used to form *descriptive summaries*. Indexing, extracting and summarising were conducted independently by two researchers. Resulting summaries were compared and discussions were held to clarify any differences. *Charting and tabulation* was conducted by charting the summaries by discipline. In order to explore similarities and differences between disciplines, we distinguished between "general medical" as papers written from a general medical practice or emergency services perspective; "psychiatry" as those written by clinical psychiatrists or from a psychiatry perspective, "Nursing" as those written by practising nurses or research nurses, "Bioethics" as those in ethics sections in journals or written by researchers in medical ethics, "Ethics" as those in ethics journals or written by ethics researchers, and "Legal" as those written from a legal perspective and/or by a legal representative. *Interpretation* of the data was conducted by thematic analysis of the summary charts to highlight the main recurrent and most important themes.[18] Two researchers conducted the thematic analysis independently and then discussed and finalised themes. Saturation of the themes was established when no further themes emerged and themes could not be further collapsed. "Vote counting" was used to identify the frequency with which the themes appeared in the selected papers.[20] In the thematic framework analysis all selected studies were included; those involving a factual case and those involving a fictional case, because both involved discussions of concerns, challenges and rationale for decision making relating to management of an advance decision following suicidal behaviour.

Quality assessment

The papers mostly comprised accounts of clinical cases written by clinicians and ethical or legal experts. The methodology quality and synthesis of case series and case reports tool suggested by Murad and colleagues[21] was used to assess the quality of selected studies. Each study was assessed independently across 4 areas of potential bias: selection, ascertainment, causality and reporting. The tool consisted of 5 items each requiring a binary

For peer review only - http://bmjopen.bmj.com/site/about/guidelines.xhtml

response to indicate whether the bias was likely. We considered the quality of the study good when all five criteria were fulfilled, moderate when 4 were fulfilled and poor when 3 or less were fulfilled. The methodological quality of included studies was assessed independently by two reviewers and discussion between them where there was disagreement. We also considered the reflexivity of the author/s, their expertise and how they were involved in the clinical case (for example as a clinician or legal/ethics consultant). Authors of the papers reflected on the management of the clinical case, rationale for decision made and issues relating to advance decisions and suicidal behaviour more generally.

Patient and Public Involvement

An expert by-experience was a co-applicant on the NIHR Programme Grant and actively contributed to the study design and objectives. Patient advisors, carers, and clinicians evaluated the relevance and importance of the research questions for the advance decisions component of the Grant and the systematic review. Our interim and final results were presented and evaluated by clinicians, academics, patients, and carers. There was also patient input into our dissemination plan, which includes dissemination to clinicians and the relevant patient community.

Results

Systematic search

Results of the systematic search are displayed in Figure 1. After removal of duplicates, the search returned 634 articles, of which 35 were retained after screening based on title/abstract. Following full text screening, 15 articles were retained for data extraction.
Study Characteristics

Descriptive information about the selected articles is displayed in Table 3. Five of the selected articles were from the UK and the others were from the US (n = 7) or Australia (n =

For peer review only - http://bmjopen.bmj.com/site/about/guidelines.xhtml

3). A total of six clinical cases were reviewed across the 15 articles (see Table 3), as seven (47%) of the articles reported the same case (Case A, a well-publicised case of a 26 year old woman who died in the UK). Two of the clinical cases presented fictional scenarios.[2, 22]

Study quality assessment

All 15 studies were assessed for bias using the methodology quality and synthesis of case series and case reports tool suggested by Murad and colleagues.[21] Nine of the selected studies were deemed to have moderate methodologic quality and 6 to have poor quality (see supplementary information 2). The quality assessment is supplied as supplementary information (Supplementary Information 2). None of the studies reported the representativeness or selection process relating to the case report, which impacted on the bias ratings. Although case reports are considered to have increased risk of bias, they have profoundly influenced medical literature and advance knowledge and their use in reviews is considered appropriate where no other higher level evidence is available.[21]

Textual synthesis

Examination of clinical cases discussed in the selected articles

Specific information about clinical cases and decision-making is summarised and charted in Table 4. We only included examination of the factual cases (n = 6) in this part of the analysis, because we were interested in the types of real-world cases and decisions made, rather than an examination of a hypothetical scenario.

Patients discussed in the clinical cases varied in age, ranging from 26-86 years old. All patients were noted as having a diagnosis of depression, some were reported as also having diagnoses of Post-traumatic stress disorder and personality disorders. The suicide methods used in the cases included self-poisoning (n = 3), gunshot incidents (n =2) and hanging (n =1). All patients were found by other people, except one patient who called an ambulance

For peer review only - http://bmjopen.bmj.com/site/about/guidelines.xhtml

because they did not want to die alone. Four of the patients were reported to have died; the outcome in one case was not specified.

Treatment was provided in only one of the clinical scenarios.[23] In this case, the patient was a psychiatric inpatient and the advance decision was considered part of the suicide attempt, so the patient's treatment refusal specified in the advance decision document was not adhered to.

The rationale for non-treatment in the clinical cases where the patient died varied and was summarised into the following three reasons:

- Advance decision was followed as a *legally-binding document* after checks showed the information was clear and specific, patient was informed of treatment options, had mental capacity at the time of writing and family were in agreement with the decision for non-treatment (n = 1).[8, 24]
- Physical injuries were severe resulting in *poor prognosis* for the patient and the treatment refusal in the advance decision was used as evidence that the patient would not wish to survive with a life-threatening or severely disabling condition. Where possible, families were also consulted (n = 2).[7, 25]
- *Verbal treatment refusal* was used as the basis for the treatment decision, rather than the advance decision, because the patient was conscious and had mental capacity. Consultation with family was not reported in this case. (n =1).[6, 26, 27, 28-30, 31]

The decision-making process was reported to take considerable time and legal and/or ethical consultation took place in all the reported clinical cases.

Differences in opinions about clinical management and decision-making between emergency department clinicians and psychiatric consultants were reported in some of the clinical cases.[23, 25] In those cases, emergency department clinicians gave more weight to the advance decision, suggesting it should be adhered to as a legally binding document and the patient remain untreated. In contrast psychiatrists viewed suicide as a consequence of a

For peer review only - http://bmjopen.bmj.com/site/about/guidelines.xhtml

distressed state and expressed a preference to avoid adherence with the advance decision and treat the patient. Where such conflict arose this was resolved through consultation with the hospital legal team and/or ethics committee.

Thematic analysis

Five themes arose from the thematic analysis and are presented with their corresponding sub-themes and vote-counts in Table 5. We included accounts of fictional cases in the thematic analysis because here we were interested in opinions, views and perspectives of authors.

Themes

1) Tension between patient autonomy and protecting a vulnerable person

Professional dilemma: promoting patient autonomy vs. providing appropriate care

The management of an advance decision in the context of suicidal behaviour was particularly challenging because it went against healthcare professionals' training to preserve life (i.e. adherence to the advance decision could result in the death of the patient while they could recover if they received treatment for their physical condition). This presented clinicians with a dilemma between promoting patients' autonomy by observing their wishes stated in the advance decision and by providing care that was considered in their best interests (e.g. promoting life).[7, 23, 26, 28, 30]

Societal expectation to protect vulnerable person and prevent suicide

Authors also raised the issue that clinicians not only had a professional interest in protecting a vulnerable person, but there was also a societal expectation that suicide should be prevented.[23, 25, 30]

For peer review only - http://bmjopen.bmj.com/site/about/guidelines.xhtml

> “While the right to autonomy is strong, in some circumstances there may be competing rights and interests that are sufficient to override a competent decision to refuse treatment. These may include the state’s interests in preventing suicide.”[30]

The challenge to clinicians was highlighted by an acknowledgement from some authors that adherence to the advance decision in this context was emotive and would feel like assisting suicide.[24, 30]

2) Appropriateness of advance decisions for suicidal behaviour

Mental health symptoms and suicidal ideation fluctuate

Concerns were expressed about whether an advance decision should apply in the context of suicidal behaviour because of patients’ distressed state, the potential for suicidal ideation to fluctuate and for treatment preferences to change in the future.[7, 8, 31, 32]

> “The compelling notion that people will change their minds contradicts the primacy of patient autonomy in the consideration of suicide. This is what distinguishes an impulsive suicide attempt from other informed choices to obtain or refuse medical treatment by patients.”[7]

Authors from a psychiatric perspective, in particular, viewed suicidal behaviour as a symptom of a mental health condition that was potentially treatable with psychiatric care.[25] They also expressed concerns about the capacity of a distressed suicidal person to fully comprehend their decision and consider all treatment options available to them.[2, 24, 25, 32] Therefore, it was suggested by some authors that a higher level of mental capacity may be required at the time of writing the advance decision for clinicians to be confident in following it.[8] However, other authors argued that the advance decision should be considered as part of the suicide attempt and as evidence of distressed/disordered

For peer review only

thinking,[8, 23, 27, 28] rather than independently of the attempt, and the treatment refusal in the advance decision document should not be adhered to.

Advance decisions for mental and physical health conditions – are they the same?

The difference between an advance decision for suicidal behaviour and for a physical condition was highlighted across the selected papers.[6, 32] Authors from a legal perspective highlighted that the primary aim of an advance decision relating to a suicide attempt is to end life, whereas an advance decision for a chronic or terminal illness is often concerned with managing pain and avoiding prolonged suffering.[6]

There was also debate about the extent to which mental suffering legitimised suicide.[32] Authors from an ethical perspective argued that, typically, healthcare services may be more sympathetic to "end of life" decisions relating to terminal physical health conditions than mental health conditions, thus mental health patients do not receive the same palliative care options as patients without mental health diagnoses.[24] There was some discussion that it should not be assumed that psychiatric pain is more tolerable than physical pain and that both should be considered as having a similar influence on the patient.[24, 25]

3) Uncertainty about the application of legislation

Confusion and anxiety about litigation

Authors from general medical and psychiatry perspectives expressed confusion about legislation and anxiety about litigation,[2, 23, 30] with one stating that the advance decision document needed to be *'watertight'* to be considered evidence of the patient's.[25] Authors recommended that clear hospital policies be developed for advance decisions in this particular context to overcome the confusion and anxiety about ligation.[23]

> "In addition to the clinical demands associated with treating a patient with a life-threatening condition, clinicians must do their best to ascertain the patient's

For peer review only - http://bmjopen.bmj.com/site/about/guidelines.xhtml

capacity for his or her apparent decision, consider the correct ethical course, and navigate through uncharted legal waters."[7]

Authors from the UK and Australia highlighted the difficulties in implementing both mental health and mental capacity legislation when managing advance decisions relating to suicidal behaviour.[27, 29, 30, 31] Clinicians needed to consider whether someone who had attempted suicide was suffering with a mental health condition, for which they should be treated against their will. They also needed to judge whether the person had the capacity to make a decision about their treatment and, if so, that the advance decision could apply following verification checks. Some suggested that application of each legislation model (i.e. mental health or mental capacity), in isolation of the other, could result in different outcomes for the patient.[6] Some authors suggested that the difficulty with balancing mental capacity legislation and mental health legislation could be resolved by developing a single legislation that combines both.[8, 27]

Advance decisions are about more than a simple assessment of capacity

A reliance on judging a person's capacity to make a decision in the context of suicidal behaviour was discussed in detail.[8, 22, 24] The capacity assessment was discussed in relation to when the patient was involved in advance care planning and making the decision to write an advance decision to refuse treatment.[8] Capacity assessment was also discussed in relation to clinicians in an emergency situation, when if the person is considered to have capacity the advance decision can be ignored and they can verbally refuse/accept treatment. While this is an important part of some legislation, particularly in the UK, it was suggested that an assessment of capacity should be supplemented with a judgment of the authenticity and durability of the patient's decision (i.e. if the decision had been consistent over time).[22, 26] Authors from a psychiatric perspective, in particular, suggested that advance decisions should be regularly reviewed to ensure that they were up-to-date and continued to reflect the patient's desires and preferences.[26, 27, 28]

For peer review only - http://bmjopen.bmj.com/site/about/guidelines.xhtml

4) The length of time needed to consider all the evidence vs. rapid decision-making for treatment

Need to fully consider the totality of evidence

Some authors suggested that the increased length of time taken in this particular context arose from the need to consider contextual factors for the suicidal behaviour,[2, 22, 25] the patient's mental health background[27] and the reason for their decision, alongside the usual validation checks and judgment as to presence of mental capacity at the time of making the advance decision. It was also argued that clinicians should take into account wider factors that may have not been present when the person first wrote the advance decision, such as changes in evidence-base for a particular treatment or scientific advances offering new treatment options that may influence the patient's decision.[22]

However, authors highlighted difficulties with gaining access to such evidence, particularly in emergency situations, further adding to the time taken to make a decision.[31] It was noted that advance decisions were often too specific (e.g. related to a specific illness or injury) or too general (e.g. a general refusal of treatment, rather than refusal of a specific treatment), resulting in ambiguity as to the best course of action for the patient and time consuming investigation.[2,25,28] Some authors highlighted that advance decisions were not useful in emergency settings when rapid decision-making was required.[2] Advance decisions may be more appropriate for patients to express refusals of on-going psychiatric treatment (e.g. Electroconvulsive therapy).

Increased gravity of the clinical decision

Authors argued that the gravity of the clinical decision was increased in this context because the patient could die if the advance decision was adhered to when recovery from mental ill health may be possible.[6, 25] Authors suggested that validation checks in this context may need to be more thorough and authors from a legal perspective argued that, because of the increased gravity of the clinical decision, physicians should seek a consensus about clinical

For peer review only - http://bmjopen.bmj.com/site/about/guidelines.xhtml

management, whilst providing life-sustaining treatment, creating a time-consuming situation.[7, 31]

5) Importance of seeking support and sharing the decision

Drawing up an advance decision as a collaborative process

Some authors argued that when writing an advance decision, patients should be supported by a healthcare professional to consider all possible treatment options.[2, 22, 23, 27, 29] It was suggested that evidence of mental capacity at the time of writing the advance decision should be provided (e.g. verified and signed by the healthcare professional) which could help with clinical decision-making at a later stage.[22] Authors from all the perspectives stressed the importance of also consulting with a physician at the time of writing the advance decision to ensure that it is both specific and general enough to be helpful and informative in a given medical scenario.[23, 27]

Shared decision making

All authors discussed the need for multi-agency decision-making in relation to the management of advance decisions in the context of suicidal behaviour.[7, 27, 28] Suggestions included that clinicians should consult widely, make use of psychiatric expertise, review the patient's psychiatric history and background and seek legal and/or ethical consultation when considering treatment decisions.

Discussion

Summary of the findings

A comprehensive systematic review of studies examining the management of advance decisions to refuse treatment following suicidal behaviour was conducted. The findings show a paucity of studies in this specific area. Fifteen relevant studies were identified, of which all were reports of clinical cases. With the exception of two papers that noted

For peer review only - http://bmjopen.bmj.com/site/about/guidelines.xhtml

fictional clinical cases, the others reported on six real clinical cases. Despite having no language or country restrictions to the search, all the studies were from the US, Australia or UK, which have similar legislation relating to advance care planning and advance decisions to refuse treatment.[2]

There were inconsistent views on practice and rationales for the management of advance decisions. Treatment was provided in only one clinical case where the patient was a psychiatric inpatient and the advance decision was considered part of the suicide attempt.[23] In this case the patient survived and later regretted the suicide attempt. In the other clinical cases, treatment was not provided, but rationale for non-treatment differed. Rationale for treatment varied from feeling that the advance decision was legally binding[8, 24] to using the advance decision as an aide to understand the patients' treatment preferences when there was a poor prognosis or a resulting severely disabling condition.[7, 25]

Conflict between clinicians was reported in some of the reports.[23, 25] In the studies where there were conflicts, there were differences in opinions on treatment between emergency department clinicians and psychiatrists. Consultations with mental health care staff were typically sought when a patient presented with an advance decision following suicidal behaviour. Psychiatrists tended to stress the treatable nature of a mental health condition and that the suicidal behaviour was part of the mental health condition. In contrast, emergency department clinicians argued that the advance decision document was legally binding and expressed anxieties about litigation. These differences in opinion about treatment were overcome through consultations with legal and ethical representatives.

The appropriateness of advance decisions with suicidal behaviour was questioned. The questioning of the appropriateness centred largely around two reasons. First, suicide ideation was considered to fluctuate and people could change their mind about their desire to die.[7, 8, 31, 32] Although suicide has been linked to impulsivity,[33, 34] studies show that not all suicides are impulsive.[35] However, recent studies using ecological momentary assessment have shown that suicide ideation varies over short periods of time (i.e. there are changes between hours and days)[36] and follow up studies with suicide survivors tend to

For peer review only

acknowledge that they regret the suicide attempt.[37] Second, outcomes for treatment refusal following suicidal behaviour were noted to be potentially different to those for a terminal physical health condition (i.e. the patient could die when there is potential for recovery in the future). [6, 32]

Authors discussed concerns that management of advance decisions following suicidal behaviour may need to be different and present a unique clinical presentation. Similar to findings in this review, anxieties and confusion about legislation relating to advance decisions is also found in studies examining end of life care.[38] However, what does seem to differ is opinions about adherence to the advance decision to refuse treatment for chronic or terminal conditions and sympathy for assisted suicide in end of life care. Healthcare workers report support for assisted suicide relating to end of life care[39] and frustrations with continuing life-sustaining treatment where withdrawing treatment might be considered in the best interest of the patient when they have a life-threatening condition.[23, 40] Those findings indicate quite a contrast with opinions in this review where the focus was on management of advance decisions following suicidal behaviour and an expression of sympathy with the decision was not found. It will be important in future research to examine these differences further by contrasting views on management of advance decisions to refuse treatment following suicidal behaviour for patients with chronic and/or terminal physical conditions and patients who have mental health conditions without chronic or terminal physical conditions.

Management of the advance decision was difficult both emotionally and ethically for some clinicians because it challenged their professional training and their desire to protect vulnerable patients from suicide. The competing pressures of respecting a patient's right to autonomy while protecting them from the effects of mental disorder found in the current study are a commonly reported dilemma.[41] There is evidence from the present study that support for the right to autonomy may be more dominant in clinicians from emergency medicine disciplines, with those from a psychiatric background prioritising prevention of suicide. A 'middle ground' between these views may help to provide guidance for clinicians.

For peer review only - http://bmjopen.bmj.com/site/about/guidelines.xhtml

For example, in English law, courts have acknowledged that while some suicidal individuals may have capacity, the overwhelming likelihood is that capacity is impaired to at least some degree.[41] Suicidal ideation has been associated with disordered and impulsive decision making[33, 34] and evidence indicates that most mental health patients presenting to emergency departments are judged as not having capacity to make a treatment decision.[11]Therefore a higher degree of certainty should be required when assessing capacity with suicidal behaviour and clinicians should err on the side of caution .[8] Another potential resolution to this dilemma, particularly in emergency scenarios, may be to provide 'temporary intervention' to allow time for individuals to be assessed and treatment options to be discussed.[41]

An added pressure for clinicians in the management of advance decisions following suicidal behaviour was that they felt there was a societal expectation that suicide should be prevented. Adhering with the advance decision made by the patient by not treating them, not only was seen to go against their professional training to protect the patient, but it was viewed that this may be considered from a society perspective as unacceptable. The dilemma here is that a clinical decision of non-treatment and adherence with the advance decision might be accepted legally, but not socially. Concerns were expressed that this particular presentation of an advance decision met conditions that warranted overriding patients' autonomy because non-adherence with the advance decisions results in prevention of suicide, maintenance of the integrity of the medical professional and preservation of life.[25]

Recommendations for practice

Decisions made about advance decisions in the context of suicidal behaviour should be made in full consultation with psychiatric teams and with relevant legal and/or ethical advisers. The results also highlight the importance of allocating sufficient time to consider contextual evidence relating to the suicidal behaviour, the authenticity of the treatment decision and verification of the documentation/decision. Given the gravity and emotive

For peer review only - http://bmjopen.bmj.com/site/about/guidelines.xhtml

nature of a decision in this context, emergency healthcare workers may need increased support and supervision for such incidents.

Findings indicate that it may be helpful, in this particular context, for an advance decision to be written in consultation with a professional healthcare worker and the patient's family. This practice would also ensure that the patient is supported to consider all treatment options, that the advance decision is specific and detailed enough to be useful in an emergency situation and that patients' capacity at the time of writing the advance decision can be assessed and verified. The advance decision should be regularly reviewed and updated to ensure that it reflects the patient's current treatment decisions.

Strengths and Limitations

A strength of this review is that a broad range of articles from different disciplines were included, thus increasing the generalisability of results. However, there were some potential biases in the literature. First, there was a paucity of evidence: only six clinical cases were reported across the selected articles. There was also a risk of bias from the studies themselves, given that they were reviews of single clinical cases. Second, the articles were focussed on the US, UK and Australia, so may have resulted in bias relating to the specific legislation/ethics of those countries. There may be different views on this topic and its management in countries with different implementation of legislation, so it will be important for future research to compare findings internationally across a wider range of countries.[42-44] Third, as with any syntheses of qualitative data there was potential for bias to be introduced by the research team at the stages of study identification, data extraction and synthesis. This was minimised in the current study by having two researchers carry out these tasks independently and cross-check the findings.

For peer review only

Future directions

Empirical studies, such as interviews and focus groups with clinicians and patients and/or a national clinical survey are important future priorities. Given that the presentation of an advance decision following suicidal behaviour is rare, case reports are likely to continue to be important sources of information in the future and authors should be mindful to ensure that case reports include details about how information about the case were obtained and how representative it is of other cases in this area. Research examining the prevalence of advance decisions relating to suicidal behaviour could shed light on the frequency of such presentations. Suitable platforms for storing advance decisions could also be explored. For example, some have suggested a web application ('app') could better reflect the dynamic nature of treatment refusal[45] and make updating and reassessment easier.

Conclusion

Current literature on the management of advance decisions and suicidal behaviour centres on detailed accounts of clinical cases and demonstrates variability in practice and the rationale behind clinical decisions. Challenges in managing advance decisions specific to suicidal behaviour were evident and there was some debate about whether advance decisions in the context of suicidal behaviour were appropriate in their current form. Taking time to consider all the evidence when making a decision, consulting fully with mental health clinicians and seeking legal and/or ethical advice may help with some of these challenges. The support of a relevant healthcare professional at the time of writing the advance decision may also be useful.

For peer review only

References

1. Department of Health. Mental Capacity Act. London: Stationery Office, 2005.
2. Byrne, M. The use of advance directives. The use of advance directives. 2002. Nursing Monograph, 13-16.
3. Campbell LA, Kisely SR. Advance treatment directives for people with severe mental illness. The Cochrane Library. 2009 Jan 1. doi: 10.1002/14651858
4. Swanson JW, Swartz MS, Elbogen EB, Van Dorn RA, Ferron J, Wagner HR, McCauley BJ, Kim M. Facilitated psychiatric advance directives: a randomized trial of an intervention to foster advance treatment planning among persons with severe mental illness. American Journal of Psychiatry. 2006 Nov;163(11):1943-51. doi: 10.1176/ajp.2006.163.11.1943
5. Morriss R, Mudigonda M, Bartlett P, Chopra A, Jones S. National survey and analysis of barriers to the utilisation of the 2005 mental capacity act by people with bipolar disorder in England and Wales. Journal of Mental Health. 2017 Jun 19:1-8. 10.1080/09638237.2017.1340613
6. Dresser, R. Suicide attempts and treatment refusals. 2010. Hastings Centre Report, 40(30), 10-11.
7. Frank, C. How to reconcile advance care directives with attempted suicide. The Colorado Lawyer. 2013;42(7):97-101.
8. Kapur, N, Clements, C, Bateman, N, Foex, B, Mackway-Jones, K, Huxtable, R, Gunnell, D, Hawton, K. Advance directives and suicidal behaviour. British Medical Journal. 2010; 341, 590-91.
9. Hawton K, Bergen H, Casey D, Simkin S, Palmer B, Cooper J, Kapur N, Horrocks J, House A, Lilley R, Noble R. Self-harm in England: a tale of three cities. Social psychiatry and psychiatric epidemiology. 2007 Jul 1;42(7):513-21. doi: 10.1007/s00127-007-0199-7
10. Carroll R, Metcalfe C, Gunnell D. Hospital presenting self-harm and risk of fatal and non-fatal repetition: systematic review and meta-analysis. PLoS One. 2014 Feb 28;9(2):e89944. doi: 10.1371/journal.pone.0089944
11. Jacob R, Clare IC, Holland A, Watson PC, Maimaris C, Gunn M. Self-harm, capacity, and refusal of treatment: implications for emergency medical practice. A prospective observational study. Emergency Medicine Journal. 2005 Nov 1;22(11):799-802. doi: 10.1136/emj.2004.018671
12. Kapur N, Clements C, Bateman N, Foëx B, Mackway-Jones K, Hawton K, Gunnell D. Self-poisoning suicide deaths in England: could improved medical management contribute to suicide prevention?. QJM: An International Journal of Medicine. 2010 Aug 4;103(10):765-75. doi: 10.1093/qjmed/hcq128
13. Brinkman-Stoppelenburg A, Rietjens JA, van der Heide A. The effects of advance care planning on end-of-life care: a systematic review. Palliative medicine. 2014 Sep;28(8):1000-25. doi: 10.1177/0269216314526272
14. Houben CH, Spruit MA, Groenen MT, Wouters EF, Janssen DJ. Efficacy of advance care planning: a systematic review and meta-analysis. Journal of the American Medical Directors Association. 2014 Jul 31;15(7):477-89. doi: 10.1016/j.jamda.2014.01.008
15. Kapur N, Cooper J, O'Connor RC, Hawton K. Non-suicidal self-injury v. attempted suicide: new diagnosis or false dichotomy? British Journal of Psychiatry.

For peer review only - http://bmjopen.bmj.com/site/about/guidelines.xhtml

2013;202(5):326-8. doi: 10.1192/bjp.bp.112.116111. PubMed PMID: WOS:000318839900004.
16. Moher D, Shamseer L, Clarke M, Ghersi D, Liberati A, Petticrew M, Shekelle P, Stewart LA. Preferred reporting items for systematic review and meta-analysis protocols (PRISMA-P) 2015 statement. Systematic reviews. 2015 Dec;4(1):1. doi: 10.1186/2046-4053-4-1
17. Shamseer L, Moher D, Clarke M, Ghersi D, Liberati A, Petticrew M, Shekelle P, Stewart LA. Preferred reporting items for systematic review and meta-analysis protocols (PRISMA-P) 2015: elaboration and explanation. Bmj. 2015 Jan 2;349:g7647. doi. 10.1136/BMJ.g7647.
18. Centre for reviews and dissemination, Undertaking systematic reviews of research on effectiveness, CRD's guidance for carrying out or commissioning reviews, 2009, York, University of York, 3
19. Spencer L, Ritchie J. Qualitative data analysis for applied policy research. In Analyzing qualitative data 2002 Sep 9 (pp. 187-208). Routledge.
20. Ryan R; Cochrane Consumers and Communication Review Group. Cochrane Consumers and Communication Review Group: data synthesis and analysis'. http://cccrg.cochrane.org, June 2013 (accessed 25th April 2018).
21. Murad MH, Sultan S, Haffar S, et al. Methodological quality and synthesis of case series and case reports BMJ Evidence-Based Medicine Published Online First: 02 February 2018. doi: 10.1136/bmjebm-2017-110853
22. Mitchell, M. An analysis of common arguments against advance directives. 2011. Nursing Ethics, 19 (2) 245-51.
23. Cook R, Pan P, Silverman R, Soltys SM. Do-not-resuscitate orders in suicidal patients: Clinical, ethical, and legal dilemmas. Psychosomatics: Journal of Consultation and Liaison Psychiatry. 2010;51(4):277-82.
24. Sontheimer D. Suicide by advance directive? Journal of Medical Ethics. 2008;34(9):e4-e 1p.
25. Chalfin DB, Crippen D, Franklin C, Kelly DF, Kilcullen JK, Streat S, et al. 'Round-table' ethical debate: Is a suicide note an authoritative 'living will'? Critical Care. 2001;5(3):115-24.
26. Callaghan, S. & Ryan, C. J. Refusing medical treatment after attempted suicide: Rethinking capacity and coercive treatment in light of the Kerrie Wooltorton case. Journal of Law and Medicine. 2011. 18 (4) 811-819
27. David A, Hotpur, M, Mora, P, Owen, G, Szmukler, G, Richardson, G. Mentally disordered or lacking capacity? Lessons for managing serious deliberate self-harm: Advance directives and suicidal behaviour. British Medical Journal. 2010;341, 587-589.
28. Muzaffar S. 'To treat or not to treat'. Kerrie Wooltorton, lessons to learn. Emergency Medicine Journal. 2011;28(9):741-4.
29. Richardson, G. Mental capacity in the shadow of suicide: What can the law do? International Journal of Law in Context, 2013, 9 (1), 87-105.
30. Ryan, C., J. & Callaghan, S. C. Legal and ethical aspects of refusing medical treatment after a suicide attempt: the Wooltorton case in the Australian context. Clinical Practice. 2010: 193 (4) 239-243.
31. Szawarski P. Classic cases revisited: The suicide of Kerrie Wooltorton, The Intensive Care. 14: e4

For peer review only

32. Volpe, R. L., Levi, B. H., Blackall, G. F., & Green, M. J. 2012. Exploring the limits of autonomy. Hastings Centre Report, 16-18.
33. Verdier R, Castelnau D, Malafosse A, Courtet P. Impaired decision making in suicide attempters. American Journal of Psychiatry. 2005 Feb 1;162(2):304-10.
34. Dombrovski AY, Hallquist MN. The decision neuroscience perspective on suicidal behavior: evidence and hypotheses. Current opinion in psychiatry. 2017 Jan;30(1):7.
35. Baca–Garcia E, Diaz–Sastre C, Resa EG, Blasco H, Conesa DB, Oquendo MA, Saiz-Ruiz J, De Leon J. Suicide attempts and impulsivity. European archives of psychiatry and clinical neuroscience. 2005 Apr 1;255(2):152-6
36. Kleiman EM, Turner BJ, Fedor S, Beale EE, Huffman JC, Nock MK. Examination of real-time fluctuations in suicidal ideation and its risk factors: Results from two ecological momentary assessment studies. Journal of abnormal psychology. 2017 Aug;126(6):726.
37. Rosen DH. Suicide survivors: a follow-up study of persons who survived jumping from the Golden Gate and San Francisco-Oakland Bay Bridges. Western Journal of Medicine. 1975 Apr;122(4):289.
38. Meisel A, Snyder L, Quill T, American College of Physicians-American Society of Internal Medicine End-of-Life Care Consensus Panel. Seven legal barriers to end-of-life care: myths, realities, and grains of truth. Jama. 2000 Nov 15;284(19):2495-501.
39. Scherer Y, Jezewski MA, Graves B, Wu YW, Bu X. Advance directives and end-of-life decision making survey of critical care nurses' knowledge, attitude, and experience. Critical Care Nurse. 2006 Aug 1;26(4):30-40.
40. Solomon MZ, O'Donnell L, Jennings B, Guilfoy V, Wolf SM, Nolan K, Jackson R, Koch-Weser D, Donnelley S. Decisions near the end of life: professional views on life-sustaining treatments. American Journal of Public Health. 1993 Jan;83(1):14-23.
41. Allen N. The right to life in a suicidal state. International journal of law and psychiatry. 2013 Sep 1;36(5-6):350-7. doi: 10.1016/j.ijlp.2013.09.002Blank RH. End-of-life decision making across cultures. The Journal of Law, Medicine & Ethics. 2011 Jun 1;39(2):201-14. doi: 10.1111/j.1748-720X.2011.00589.x
42. Blank RH. End-of-life decision making across cultures. The Journal of Law, Medicine & Ethics. 2011 Jun 1;39(2):201-14. doi: 10.1111/j.1748-720X.2011.00589.x
43. Mishara BL, Weisstub DN. The legal status of suicide: A global review. International journal of law and psychiatry. 2016 Jan 1;44:54-74. doi: 10.1016/j.ijlp.2015.08.032
44. Van Wijmen MP, Rurup ML, Pasman HR, Kaspers PJ, Onwuteaka-Philipsen BD. Advance directives in the Netherlands: An empirical contribution to the exploration of a cross-cultural perspective on advance directives. Bioethics. 2010 Mar 1;24(3):118-26. doi: 10.1111/j.1467-8519.2009.01788.x
45. Huxtable R. Advance decisions: worth the paper they are (not) written on?. End of Life Journal. 2015 Sep 1;5(1):e000002.doi:10.1136/eoljnl-2015-000002

For peer review only - http://bmjopen.bmj.com/site/about/guidelines.xhtml

Table 1. Search terms for each topic

Advance directives	*OR*	Mental capacity	*AND*	Suicidal behaviour
advance decisions		mental		Suicide
advance directives		competency		attempted suicide
advance statement		mental capacity		self-mutilation
living will(s)				self-harm
mental health directive				deliberate self-harm
Ulysses contract(s)				parasuicide
psychiatric will(s)				self-injurious behaviour
antecedent decision/wish				drug overdose
pre-emptive suicide				self-immolation
antecedent refusal				self-poisoning
resuscitation order				self-destructive behaviour
health care power of attorney				auto aggression
				automutilation

For peer review only

Table 2. PICOS criteria for inclusion and exclusion

Parameter	Inclusion criteria	Exclusion criteria
Patients	Patients over 18 years who present to hospital with advance decisions* (also include Do Not Resuscitate orders, DNRs) following suicidal behaviour (including attempted suicide, deliberate self-harm, self-injurious behaviour, drug overdose, self-poisoning, self-destructive behaviour) with no existing chronic or terminal physical conditions	Patients who present to hospital with advance decisions but with primary conditions which were not mental health related e.g. HIV/AIDS, chronic physical health conditions or disabilities, neurodegenerative diseases and/or specific patient groups e.g. mother/baby.
Intervention	medical management and/or medico legal and/or ethical consultation/discussion	medical management of euthanasia, assisted suicide, end of life, wills/inheritance (i.e. monetary or property issues)
Comparator		
Outcomes	Adherence/non-adherence with advance decision, treatment, patient outcome (i.e. death)	
Study design	Opinion and review articles, Case studies, Empirical studies/surveys	Book reviews, Reponses to articles, conference abstracts

** or other terms such as advance decisions, advance directives, advance statement, living will(s), mental health directive, Ulysses contract(s), psychiatric will(s), mental competency, mental capacity, health care power of attorney, antecedent decision/wish, pre-emptive suicide, antecedent refusal, resuscitation order or living will, advance directive, Ulysses contract*

For peer review only

Table 3. Description of selected studies

Author	Date	Country	Perspective#	Fictional/ Factual case	Case reported*
Bryne[2]	2002	Australia	Nursing	Fictional	--
Callaghan & Ryan[26]	2011	Australia	Bioethics	Factual	A
Chalfin et al[25]	2001	US, Philadelphia, New York, New Zealand	Emergency & Acute medicine/ Bioethics	Factual	B
Cook et al[23]	2010	US, Illinois	Psychiatry	Factual	C
Dresser[6]	2010	US, New York	Legal	Factual	A
David et al[27]	2010	UK	Psychiatry	Factual	A
Frank[7]	2013	US, Colorado	Legal	Factual	D
Kapur et al[8]	2010	UK	Psychiatry	Factual	E
Mitchell[22]	2011	US, San Diego	Ethical	Fictional	--
Muzaffer[28]	2011	UK	Psychiatry	Factual	A
Richardson[29]	2013	UK	Legal	Factual	A
Ryan & Callaghan[30]	2010	Australia	Psychiatry	Factual	A
Sontheimer[24]	2008	US, Springfield	Bioethics	Factual	E
Szawarski[31]	2013	UK	Bioethics	Factual	A
Volpe et al[32]	2012	US, New York	Bioethics	Factual	F

Note: *For specific details about each case see Table 4, note fictional cases have not been given a case report ID
#where the perspective is not clearly stated this has been derived from the author(s) background and professional experience

For peer review only - http://bmjopen.bmj.com/site/about/guidelines.xhtml

Table 4. Description of clinical cases discussed in selected studies

Case	Reference	Age	Mental health conditions	Nature of SA	Resulting Injuries/ illness	Hospital admittance	Nature of the AD	When written?	Patient conscious ?	Decision Making process	Rationale for decision	Outcome
A	6, 26, 27, 28-30, 31	26	Depression generalised anxiety disorder, PTSD, BPD	Self-poisoning (anti-freeze)	Not stated	Presented herself at hospital	Letter	3 days prior	Yes	Medical staff discussed the patient's mental capacity and sought legal advice.	The patient's wishes were clear in the letter but the patient was conscious, judged to have capacity and refusing treatment.	Death
B	25	46	Severe depression	Gunshot to face	Pain and severe facial injury	Gunshot reported by neighbours	Suicide note	not stated	Yes (not coherent)	The attending physicians thought life-support should be removed as the patient's "will" was clear and authoritative. The psychiatrist thought suicide was pathological and the condition was treatable so the patient should be treated. Clinicians consulted widely and sought legal advice	The suicide note was accepted as a living will. The patient had a desire to die due to psychological pain. The suicide attempt left the patient in a severely disabled state.	Death
C	23	57	Depression generalised anxiety disorder, PTSD, BPD	Self-poisoning (opiates)	Respiratory distress	Psychiatric inpatient	DNR	Prior to inpatient admittance	Not stated	There was conflict between clinicians; the psychiatrist argued that the DNR should not be followed because it was a suicide attempt. The legal/ethics committee was consulted who supported continued treatment.	DNR considered an effort to prepare for a suicide attempt and should not be honoured.	Survived and regretted the suicide attempt.
D	7	35	Depression and drug abuse	Hanging	Brain injury	Found by family	AD	Not stated	No	There were concerns that adherence to the AD would result in the patient's death. Clinicians sought legal advice.	The patient had poor prognosis and the family gave consent for clinicians to stop treatment.	Death
E	8, 24	52	Depression generalised anxiety disorder, PTSD, BPD	Self-poisoning (insulin)	Coma	Found at home	AD	2 years prior	No	The AD mentioned no treatment for a terminal condition. The patient was not in a terminal condition and there were concerns that injury was the result of a suicide attempt and whether the AD should be adhered to in a suicidal context. Approached family and held an ethics committee consultation.	The patient's wishes were judged to be clear, the patient was considered to be informed about treatment options and had mental capacity at the time of writing the AD and the family were in agreement.	Death
F	32	86	Not stated	Gunshot to chest	Damage to pancreas and colon	Not stated	AD	Not stated	Yes (not always coherent)	Medical team argue that the nature in which the physical condition was caused (i.e. suicidal behaviour) should impact on treatment	Not stated	Not stated

Note: *for details about articles see Table 3, SA = suicide attempt, AD = advance directive, PTSD = post-traumatic stress disorder, BPD = borderline personality disorder

For peer review only - http://bmjopen.bmj.com/site/about/guidelines.xhtml

Table 5. Themes from the selected articles

Theme	Sub-themes	Theme Descriptor	Perspectives	References	Count %
Tension between patient autonomy and protecting a vulnerable person	*Professional dilemma: promoting patient autonomy vs. providing appropriate care*<br><br>*Societal expectation to protect vulnerable person and prevent suicide*	Tension between acting in accordance with patients' wishes for their medical treatment while promoting their best interests presented clinicians with a professional ethical dilemma. Clinicians also had a personal ethical dilemma, as there is societal pressure to protect vulnerable people and prevent suicide.	Psychiatry, Bioethics, Legal	7, 22, 24, 27, 29	5 (33%)
Appropriateness of advance decisions for suicidal behaviour	*Mental health symptoms and suicidal ideation fluctuate*<br><br>*Advance decisions for mental and physical health conditions – are they the same?*	There were questions about whether an advance decision "fits" in relation to suicide without an existing physical illness because mental state, mental health and suicide ideation fluctuate. Such scenarios are different from decisions made about treatment for a chronic or terminal physical condition.	Medical, Psychiatry, Bioethics, Legal	2, 6-8, 23-25, 27, 29-32	12 (80%)
Uncertainty about the application of legislation	*Confusion and anxiety about litigation*<br><br>*Advance decisions are about more than a simple assessment of capacity*	Legislation around advance decisions was seen as confusing and there was anxiety about ligation. It was noted that mental capacity legislation overlapped with mental health legislation and policy. There were concerns that relying on a capacity decision was not sufficient and the authenticity of the advance decision needed to be considered	Medical, Psychiatry, Bioethics, Legal	2, 8, 22-29, 31	11 (73%)
The length of time needed to consider all the evidence vs. rapid decision-making for treatment	*Need to fully consider the totality of evidence*<br><br>*Increased gravity of the clinical decision*	Clinical decisions were considered to be complex, involving an assessment of mental capacity, verification of the advance decision, and consideration of contextual factors. Therefore sufficient time was needed in which to consider all of the evidence.	Medical, Psychiatry, Bioethics Legal	2, 8, 25-27	5 (33%)
Importance of seeking support and sharing the decision	*Drawing up an advance decision as a collaborative process*<br><br>*Shared decision making*	Sharing the decision-making and seeking support, both at the time of writing the advance decision and when treating the patient, was viewed as important.	Medical, Psychiatry, Bioethics, Legal	2, 7, 24-28, 30, 31	9 (60%)

For peer review only - http://bmjopen.bmj.com/site/about/guidelines.xhtml

Figures

Figure 1. Flow chart of results from initial search

For peer review only

Figure 1. Flow chart of results from initial search

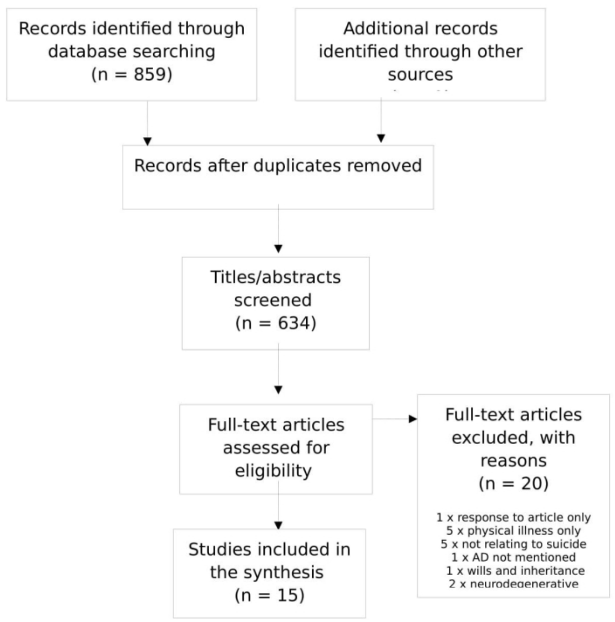

Figure 1

139x198mm (300 x 300 DPI)

Supplementary Information 1: Database Search Strategy

Psychinfo:

(((advance decision or advance directive or advance statement or living will or living wills or mental health directive or Ulysses contract or Ulysses contracts or psychiatric will or psychiatric wills or mental competency or mental capacity or healthcare power of attorney or health care power of attorney or antecedent decision or antecedent wish* or preemptive suicide or antecedent refusal or resuscitation orders)

and

(suicide or attempted suicide or self mutilation or self-harm or deliberate self-harm or self harm or parasuicid$ or para-suicd$ or "self-injurious behaviour" or drug overdose or self immolation or self poisoning or self-destructive behav or autoaggress$ or automutilia$)) not (euthanasia or assisted suicide)).af.

Pubmed:

("advance decisions" or "advance directives" or "advance statement" or "living will" or "living wills" or "mental health directive" or "ulysses contract" or "ulysses contracts" or "psychiatric will" or "psychiatric wills" or "mental competency" or "mental capacity" or "healthcare power attorney" or "healthcare power of attorney" or "antecedent decision" or "antecedent wish" or "preemptive suicide" or "antecedent refusal" or "resuscitation orders" or "do not resuscitate" or DNR order)
suicid

EBESCO:

(suicide or attempted suicide or self mutilation or self-harm or deliberate self-harm or self harm or parasuicid$ or para-suicd$ or "self-injurious behaviour" or drug overdose or self immolation or self poisoning or self-destructive behav or autoaggress$ or automutilia$).ab

and

(advance decision or advance directive or advance statement or living will or living wills or mental health directive or Ulysses contract or Ulysses contracts or psychiatric will or psychiatric wills or mental competency or mental capacity or healthcare power of attorney or health care power of attorney or antecedent decision or antecedent wish* or preemptive suicide or antecedent refusal or resuscitation orders).ab

For peer review only

EMBASE:

(advance decision or advance directive or advance statement or living will or living wills or mental health directive or Ulysses contract or Ulysses contracts or psychiatric will or psychiatric wills or mental competency or mental capacity or healthcare power of attorney or health care power of attorney or antecedent decision or antecedent wish* or preemptive suicide or antecedent refusal or resuscitation orders).ab.

and

(suicide or attempted suicide or self mutilation or self-harm or deliberate self-harm or self harm or parasuicid$ or para-suicd$ or "self-injurious behaviour" or drug overdose or self immolation or self poisoning or self-destructive behav or autoaggress$ or automutilia$).ab

MEDLINE:

(((advance decision or advance directive or advance statement or living will or living wills or mental health directive or Ulysses contract or Ulysses contracts or psychiatric will or psychiatric wills or mental competency or mental capacity or healthcare power of attorney or health care power of attorney or antecedent decision or antecedent wish* or preemptive suicide or antecedent refusal or resuscitation orders) and (suicide or attempted suicide or self mutilation or self-harm or deliberate self-harm or self harm or parasuicid$ or para-suicd$ or "self-injurious behaviour" or drug overdose or self immolation or self poisoning or self-destructive behav or autoaggress$ or automutilia$)) not (euthanasia and assisted suicide)).ab.

CINAHL

AB (advance decisions OR (advance directives and living wills) OR mental capacity OR mental competency OR health care power of attorney OR antecedent decision OR preemptive suicide OR resuscitation orders OR (dnr or do not resuscitate) OR (dnr orders and ethical principles))

AND AB suicide OR suicide attempt OR self-harm OR self harm OR deliberate self harm OR self-injurious behavior OR (self injury or self harm or self mutilation) OR drug overdose OR self immolation OR self-destructiv behaviors OR self-poisoning
AB ((advance decisions OR (advance directives and living wills) OR mental capacity OR mental competency OR health care power of attorney OR antecedent decision OR preemptive suicide OR resuscitation orders OR (dnr or do not resuscitate) OR (dnr orders and ethical principles))) AND AB (suicide OR suicide attempt OR self-harm OR self harm OR deliberate self harm OR self-injurious behavior OR (self injury or self harm or self mutilation) OR drug overdose OR self immolation OR self-destructiv behaviors OR self-poisoning) NOT AB assisted suicide NOT AB (euthanasia or assisted suicide)

For peer review only

Social Policy and Practice:

(("advance decisions" or "advance directives" or "advance statement" or "living will" or "living wills" or "mental health directive" or "ulysses contract" or "ulysses contracts" or "psychiatric will" or "psychiatric wills" or "mental competency" or "healthcare power attorney" or "healthcare power of attorney" or "antecedent decision" or "antecedent wish" or "pre emptive suicide" or "preemptive suicide" or "antecedent refusal" or "resuscitation orders" or "do not resuscitate" or "DNR order") not (euthanasia and "assisted suicide")).af.

and

(suicide or "attempted suicide" or "self-mutilation" or "deliberate self-harm" or "self-harm" or Parasuicide or Suicid* or "drug-overdose" or "self-poisioning" or "self-immolation" or "suicidal behav*" or "self-destructive behav*" or Autoaggress$ or "self-injurious behav*" or "non suicidal self-injury" or "non fatal self-harm" or "completed suicide" or automutilla$).af

For peer review only

Supplementary Information 2: Quality Assessment of studies

Author	Question 1		Question 2		Question 3		Question 4		Question 5		Methodological quality
	Yes	No	Yes	No	Yes	No	Yes	No	Yes	No	
Bryne[2]		X		X		X		X		X	Poor
Callaghan & Ryan[22]		X	X		X		X		X		Moderate
Chalfin et al[23]		X	X			X	X		X		Poor
Cook et al[24]		X	X		X		X		X		Moderate
Dresser[6]		X		X		X	X			X	Poor
David et al[25]		X	X		X		X		X		Moderate
Frank[7]		X	X			X		X	X		Poor
Kapur et al[8]		X	X		X			X		X	Poor
Mitchell[26]		X		X		X		X		X	Poor
Muzaffer[27]		X	X			X	X			X	Poor
Richardson[28]		X	X		X		X		X		Moderate
Ryan & Callaghan[29]		X	X		X		X		X		Moderate
Sontheimer[30]		X	X		X			X	X		Poor
Szawarski[31]		X	X		X		X		X		Moderate
Volpe et al[32]		X	X			X		X		X	Poor

Note: *Selection*: question 1: Did the patient(s) represent the whole experience of the investigator or is the selection method unclear to the extent that other patients with similar presentations may not have been presented?; *Ascertainment*: question 2: Was the case adequately ascertained?, question 3: Was the outcome adequately ascertained?; *Causality*: question 4: Was follow-up long enough for outcomes to occur?; *Reporting*: question 5: Is the case described with sufficient details to allow practitioners to make inferences on their own practice?

For peer review only

PRISMA 2009 Checklist

Section/topic	#	Checklist item	Reported on page #
**TITLE**			
Title	1	Identify the report as a systematic review, meta-analysis, or both.	1
**ABSTRACT**			
Structured summary	2	Provide a structured summary including, as applicable: background; objectives; data sources; study eligibility criteria, participants, and interventions; study appraisal and synthesis methods; results; limitations; conclusions and implications of key findings; systematic review registration number.	4
**INTRODUCTION**			
Rationale	3	Describe the rationale for the review in the context of what is already known.	7
Objectives	4	Provide an explicit statement of questions being addressed with reference to participants, interventions, comparisons, outcomes, and study design (PICOS).	7
**METHODS**			
Protocol and registration	5	Indicate if a review protocol exists, if and where it can be accessed (e.g., Web address), and, if available, provide registration information including registration number.	No protocol
Eligibility criteria	6	Specify study characteristics (e.g., PICOS, length of follow-up) and report characteristics (e.g., years considered, language, publication status) used as criteria for eligibility, giving rationale.	8 (Table 1 and 2)
Information sources	7	Describe all information sources (e.g., databases with dates of coverage, contact with study authors to identify additional studies) in the search and date last searched.	8
Search	8	Present full electronic search strategy for at least one database, including any limits used, such that it could be repeated.	8 (Table 1 and 2)
Study selection	9	State the process for selecting studies (i.e., screening, eligibility, included in systematic review, and, if applicable, included in the meta-analysis).	8 (Table 1 and 2)
Data collection process	10	Describe method of data extraction from reports (e.g., piloted forms, independently, in duplicate) and any processes for obtaining and confirming data from investigators.	9
Data items	11	List and define all variables for which data were sought (e.g., PICOS, funding sources) and any assumptions and simplifications made.	9
Risk of bias in individual studies	12	Describe methods used for assessing risk of bias of individual studies (including specification of whether this was done at the study or outcome level), and how this information is to be used in any data synthesis.	10
Summary measures	13	State the principal summary measures (e.g., risk ratio, difference in means).	n/a

For peer review only

PRISMA 2009 Checklist

Synthesis of results	14	Describe the methods of handling data and combining results of studies, if done, including measures of consistency (e.g., I^2) for each meta-analysis.	9

Page 1 of 2

Section/topic	#	Checklist item	Reported on page #
Risk of bias across studies	15	Specify any assessment of risk of bias that may affect the cumulative evidence (e.g., publication bias, selective reporting within studies).	10
Additional analyses	16	Describe methods of additional analyses (e.g., sensitivity or subgroup analyses, meta-regression), if done, indicating which were pre-specified.	9
**RESULTS**			
Study selection	17	Give numbers of studies screened, assessed for eligibility, and included in the review, with reasons for exclusions at each stage, ideally with a flow diagram.	10 (Figure 1)
Study characteristics	18	For each study, present characteristics for which data were extracted (e.g., study size, PICOS, follow-up period) and provide the citations.	10-12, 21 (Table 3)
Risk of bias within studies	19	Present data on risk of bias of each study and, if available, any outcome level assessment (see item 12).	n/a
Results of individual studies	20	For all outcomes considered (benefits or harms), present, for each study: (a) simple summary data for each intervention group (b) effect estimates and confidence intervals, ideally with a forest plot.	Textual analysis 10-12
Synthesis of results	21	Present results of each meta-analysis done, including confidence intervals and measures of consistency.	Synthesis of Qual results 12-17
Risk of bias across studies	22	Present results of any assessment of risk of bias across studies (see Item 15).	Noted in discussion p19
Additional analysis	23	Give results of additional analyses, if done (e.g., sensitivity or subgroup analyses, meta-regression [see Item 16]).	n/a
**DISCUSSION**			
Summary of evidence	24	Summarize the main findings including the strength of evidence for each main outcome; consider their relevance to key groups (e.g., healthcare providers, users, and policy makers).	17-18
Limitations	25	Discuss limitations at study and outcome level (e.g., risk of bias), and at review-level (e.g., incomplete retrieval of identified research, reporting bias).	19

For peer review only

PRISMA 2009 Checklist

Conclusions	26	Provide a general interpretation of the results in the context of other evidence, and implications for future research.	20
**FUNDING**			
Funding	27	Describe sources of funding for the systematic review and other support (e.g., supply of data); role of funders for the systematic review.	2

From: Moher D, Liberati A, Tetzlaff J, Altman DG, The PRISMA Group (2009). Preferred Reporting Items for Systematic Reviews and Meta-Analyses: The PRISMA Statement. PLoS Med 6(7): e1000097. doi:10.1371/journal.pmed1000097

For more information, visit: **www.prisma-statement.org**.

Page 2 of 2

For peer review only

ENTREQ checklist (Enhancing transparency in reporting the synthesis of qualitative research) *

No. Item	Guide questions/description	Reported on Page #
1. Aim	State the research question the synthesis addresses	8
2. Synthesis methodology	Identify the synthesis methodology or theoretical framework which underpins the synthesis, and describe the rationale for choice of methodology (e.g. meta-ethnography, thematic synthesis, critical interpretive synthesis, grounded theory synthesis, realist synthesis, meta-aggregation, meta-study, framework synthesis)	10-11
3. Approach to searching	Indicate whether the search was pre-planned (comprehensive search strategies to seek all available studies) or iterative (to seek all available concepts until they theoretical saturation is achieved)	9
4. Inclusion criteria	Specify the inclusion/exclusion criteria (e.g. in terms of population, language, year limits, type of publication, study type	Table 2
5. Data sources	Describe the information sources used (e.g. electronic databases (MEDLINE, EMBASE, CINAHL, psycINFO), grey literature databases (digital thesis, policy reports), relevant organisational websites, experts, information specialists, generic web searches (Google Scholar) hand searching, reference lists) and when the searches conducted; provide the rationale for using the data sources	9
6. Electronic Search strategy	Describe the literature search (e.g. provide electronic search strategies with population terms, clinical or health topic terms, experiential or social phenomena related terms, filters for qualitative research, and search limits)	9 Supplementary Info 1
7. Study screening methods	Describe the process of study screening and sifting (e.g. title, abstract and full text review, number of independent reviewers who screened studies	9-10
8. Study characteristics	Present the characteristics of the included studies (e.g. year of publication, country, population, number of participants, data collection, methodology, analysis, research questions)	12 Table 2
9. Study selection results	Identify the number of studies screened and provide reasons for study exclusion (e,g, for comprehensive searching, provide numbers of studies screened and reasons for exclusion indicated in a figure/flowchart; for iterative searching describe reasons for study exclusion and inclusion based on modifications to the research question and/or contribution to theory development)	12-13 Figure 1
10. Rationale for appraisal	Describe the rationale and approach used to appraise the included studies or selected findings (e.g. assessment of conduct (validity and robustness), assessment of reporting (transparency), assessment of content and utility of the findings)	11-13
11. Appraisal items	State the tools, frameworks and criteria used to appraise the studies or selected findings (e.g. Existing tools: CASP, QARI, COREQ, Mays and Pope [25]; reviewer developed tools; describe the domains assessed: research team, study design, data analysis and interpretations, reporting)	11-13
12. Appraisal process	Indicate whether the appraisal was conducted independently by more than one reviewer and if consensus was required	13
13. Appraisal	Present results of the quality assessment and indicate which	13,

For peer review only - http://bmjopen.bmj.com/site/about/guidelines.xhtml

results	articles, if any, were weighted/excluded based on the assessment and give the rationale	Supplementary info 2
14. Data extraction	Indicate which sections of the primary studies were analysed and how were the data extracted from the primary studies? (e.g. all text under the headings "results /conclusions" were extracted electronically and entered into a computer software)	10-11
15. Software	State the computer software used, if any	n/a
16. Number of reviewers	Identify who was involved in the coding and analysis	10-11
17. Coding	Describe the process for coding of data (e.g. line by line coding to search for concepts)	11
18. Study comparison	Describe how were comparisons made within and across studies (e.g. subsequent studies were coded into pre-existing concepts, and new concepts were created when deemed necessary)	11
19. Derivation of themes	Explain whether the process of deriving the themes or constructs was inductive or deductive	11
20. Quotations	Provide quotations from the primary studies to illustrate themes/constructs, and identify whether the quotations were participant quotations of the author's interpretation	15-20
21. Synthesis output	Present rich, compelling and useful results that go beyond a summary of the primary studies (e.g. new interpretation, models of evidence, conceptual models, analytical framework, development of a new theory or construct)	13-20

* Reference: Tong A, Flemming K, McInnes E, Oliver SA, Craig J. Enhancing transparency in reporting the synthesis of qualitative research: ENTREQ. BMC Medical Research Methodology 2012, 12:181.

For peer review only

BMJ Open

The management of patients with an advance decision and suicidal behaviour: A systematic review

Journal:	*BMJ Open*
Manuscript ID	bmjopen-2018-023978.R2
Article Type:	Research
Date Submitted by the Author:	19-Nov-2018
Complete List of Authors:	Nowland (Harris), Rebecca; University of Manchester, Centre for Health and safety<br>Steeg, Sarah; University of Manchester, Psychiatry<br>Quinlivan, Leah; University of Manchester , Brain, Behaviour and Mental Health<br>Cooper, Jayne; University of Manchester,<br>Huxtable, Richard; University of Bristol, School of Social and Community Medicine<br>Hawton, Keith; Centre for Suicide Research, Oxford University, Psychiatry<br>Gunnell, DJ; University of Bristol, Social Medicine<br>Allen, Neil; University of Manchester, School of Law<br>Mackway-Jones, Kevin; Manchester Royal Infirmary, Emergency Department<br>Kapur, Navneet; University of Manchester, Centre for suicide prevention
**Primary Subject Heading**:	Mental health
Secondary Subject Heading:	Emergency medicine, General practice / Family practice, Medical management
Keywords:	Suicide & self-harm < PSYCHIATRY, suicidal behaviour, advance decisions, advance directives, living wills

SCHOLARONE™
Manuscripts

Title: The management of patients with an advance decision and suicidal behaviour: A systematic review

Author list: Rebecca Nowland, PhD[1]; Sarah Steeg, PhD[1]; Leah Quinlivan, PhD[1,2]; Jayne Cooper, PhD[1], Prof Richard Huxtable[3]; Prof Keith Hawton, FMedSc[4]; Prof David Gunnell DSc[5], Neil Allen (Barrister)[6], Prof Kevin Mackway-Jones[7], Prof Nav Kapur, FRPsych[1,2, 8]

1. Centre for Suicide Prevention, Manchester Academic Health Science Centre, University of Manchester, England
2. NIHR Greater Manchester Patient Safety Translational Research Centre, England
3. Centre for Ethics in Medicine, University of Bristol, England
4. Centre for Suicide Research, University Department of Psychiatry, Warneford hospital, Oxford, England
5. Department of Population Health Sciences, University of Bristol, England
6. School of Law, University of Manchester, England
7. Emergency Department, Manchester Royal Infirmary, Manchester University NHS Foundation Trust, Manchester, England
8. Greater Manchester Mental Health NHS Foundation Trust, Manchester, England.

Corresponding author: Dr Rebecca Nowland

Address: School of Nursing, Brook Building, University of Central Lancashire, PR1 2HE

Telephone: +44 (0)161 275 0727

Email: RNowland@uclan.ac.uk

Word count = 5853 (excluding abstract and references)

For peer review only

Authorship and contribution

All authors made substantial contributions to the study. RN and LQ conducted the initial scoping search. RN designed the review and data extraction/analysis with input from NK and SS. RN and SS screened and reviewed the articles and performed data extraction/analysis, interpreted the results and wrote the first draft. NK, JC, RH, KH, DG, NA, and KMJ reviewed the initial draft, contributed to subsequent drafts and approved the final version. All authors take responsibility for the integrity of the data analysis. NK is the guarantor of the study.

Declaration of Interest

DG, KH, and NK are members of the Department of Health's (England) National Suicide Prevention Advisory Group. NK chaired the NICE guideline development group for the longer term management of self-harm and the NICE Topic Expert Group (which developed the quality standards for self-harm services). He is currently chair of the updated NICE guideline for Depression. KH and DG are NIHR Senior Investigators. KH is also supported by the Oxford Health NHS Foundation Trust and NK by the Greater Manchester Mental Health NHS Foundation Trust.

Funding

This paper presents independent research funded by the National Institute of Health Research (NIHR) under its Programme Grants for Applied Research Programme (Grant Reference Number RP-PG-0610-10026). The views expressed are those of the authors and not necessarily those of the NHS, the National Institute of Health Research or the Department of Health.

Data sharing statement

No additional data are available

Provenance and peer review

Not commissioned; externally peer reviewed

For peer review only

Acknowledgements

We would to thank Rosie Davies, Research Fellow in Patient and Public Involvement at the University of Bristol, for her input into the study.

For peer review only

ABSTRACT

Background: The use of advance care planning and advance decisions for psychiatric care is growing. However, there is limited guidance on clinical management when a patient presents with suicidal behaviour and an advance decision and no systematic reviews of the extant literature.

Objectives: To synthesise existing literature on the management of advance decisions and suicidal behaviour.

Design: A systematic search of 7 bibliographic databases was conducted to identify studies relating to advance decisions and suicidal behaviour. Studies on terminal illness or end of life care were excluded to focus on the use of advance decisions in the context of suicidal behaviour. A textual synthesis of data was conducted and themes were identified by using an adapted thematic framework analysis approach.

Results: Overall 634 articles were identified, of which 35 were retained for full text screening. Fifteen relevant articles were identified following screening. Those articles pertained to actual clinical cases or fictional scenarios. Clinical practice and rationale for management decisions varied. Five themes were identified: 1) tension between patient autonomy and protecting a vulnerable person, 2) appropriateness of advance decisions for suicidal behaviour, 3) uncertainty about the application of legislation, 4) the length of time needed to consider all the evidence vs. rapid decision-making for treatment, and 5) importance of seeking support and sharing decision-making.

Conclusions: Advance decisions present particular challenges for clinicians when associated with suicidal behaviour. Recommendations for practice and supervision for clinicians may help to reduce the variation in clinical practice.

Keywords: self-harm, suicidal behaviour, advance directives, advance decisions, living wills, Ulysses directives

For peer review only

Article Summary

Strengths and limitations of this study

- Timely systematic review considering the challenges relating to advance decisions in the context of suicidal behaviour
- Review involves journal articles from a variety of countries from a range of disciplines
- Paucity of evidence for this specific presentation of advance decision
- Evidence in this area is predominately from reviews of case studies, rather than empirical work

For peer review only

INTRODUCTION

An advance decision (sometimes known as an advance decision to refuse treatment (ADRT) or living will) is typically a written document that outlines a person's desire to refuse certain treatments, including life-saving treatment, when there is a potential for a person to lose the mental capacity to make treatment decisions in the future.[1] In order for an advance decision to be valid, the person must have mental capacity at the time of writing the document. Mental capacity is defined as the ability to make a decision and involves understanding and weighing information relating to a decision and alternative options and retaining that information long enough to make the decision.[1] The Mental Capacity Act in England and Wales refers to "advance decisions to refuse treatment (ADRT)," but more widely these documents are referred to as "advance directives" and/or "living wills". We use "advance decision" throughout in this paper to refer to written documents stating a refusal of treatment made in advance of medical treatment following an illness or injury.

There are important cross-national variations in legislation; in some countries, the use of advance decisions is not permitted (i.e. Turkey, Japan), while in others, advance decisions are legislated for (i.e. the UK and US). The UK, Australia and US have similar legal standards with some state-wide variation in the US and Australia,[2] with some states adopting the common law right to make an advance decision and others allowing the use of a surrogate or proxy decision maker (i.e. to make healthcare decisions on behalf of the patient). There is also considerable variation in practice between countries where advance decisions are permitted. For example, in Germany, advance decisions are recognised but require court approval in each case.[2]

Advance care planning for psychiatric care is becoming more common in a number of countries, including the UK, US and Australia[3, 4] and enables patients to state their preferences for the management of their mental health condition when they may temporarily lose their mental capacity. A person with a mental health condition may also make some decisions about particular treatment that they would not wish to have and may involve an advance decision to refuse particular treatments, i.e. electroconvulsive therapy. Advance care planning has been shown to have a number of healthcare benefits for mental health patients

For peer review only - http://bmjopen.bmj.com/site/about/guidelines.xhtml

in the UK and US, such as enhancing patient autonomy and engagement, promoting adherence to treatment plans (i.e. patients taking prescribed drugs), improving continuity of care with fewer psychiatric admissions, reducing the use of social workers' time and lower levels of violent acts.[3,4] In a recent survey of patients with bipolar disorder, 21% had written statements about their healthcare, and of those, 10% involved an advance decision to refuse treatment.[5] This increasing use of advance care planning in mental health may result in an increasing use of advance decisions to refuse mental health care treatment, and concerns about clinical management of advance decisions following suicidal behaviour have been made by healthcare professionals and legal and ethical consultants.[6-8] Existing literature, from a variety of academic and clinical perspectives, suggests there is little consistency in practice and there are specific challenges with advance decisions following suicidal behaviour. Such scenarios raise questions about whether a person with a wish to end their life has the capacity to make a decision about refusal of treatment and/or if their capacity is affected by mental illness, and whether an advance decision is appropriate for medical treatment following suicidal behaviour.[8]

The terminology for suicidal behaviour varies internationally. Some clinicians/researchers distinguish between suicide attempts and non-suicidal self-injury[9, while others prefer the broad term of self-harm to denote behaviours across the spectrum.[1, 10] We have taken an inclusive approach in this review to ensure we captured relevant studies, so in this review we refer to "suicidal behaviour" as behaviours including all self-harming behaviour (including non-suicidal injury) and suicide attempts. The use of "suicidal behaviour" in our review means that there may be cases of non-suicidal injury that may be included.

The management of suicidal behaviour is a significant challenge for clinicians in the emergency services. Each year over 200,000 people present to emergency departments in England with self-harm,[10] with 16% of those presenting to hospital with a repeat self-harm episode within a year.[11] Treatment refusal following suicidal behaviour has been shown to be common. A prospective cohort study of mental capacity and suicidal behaviour in the Emergency Department (ED) found that around 40% of patients presenting to hospital with self-harm had the capacity to make a decision about their medical treatment and 30% of those intended to refuse life-saving treatment.[12] There are few studies that have examined

For peer review only - http://bmjopen.bmj.com/site/about/guidelines.xhtml

numbers of advance decisions to refuse treatment in patients presenting with suicidal behaviour, but in a recent study, three out of 121 fatal cases of self-poisoning in 2005, the patients had an advance decision.[13] Given that patient autonomy and advance care planning are encouraged in modern healthcare and are assuming greater prominence, it is likely that the number of people presenting to hospital with an advance decision following suicidal behaviour will grow.

Rationale

For peer review only

While reviews of literature relating to the management of advance decisions, both more broadly and specifically to relating to “end of life” care exist,[14, 15] there are currently no reviews on the management of advance decisions when a patient presents to hospital following suicidal behaviour where the patient does not have a chronic or terminal physical illness. Despite the legislative context being similar for end of life care, the ethical considerations, emotional challenges and clinical decision-making may be different for treatment of a patient following suicidal behaviour without a chronic or terminal physical illness. A synthesis of this literature is important to examine similarities and differences and to establish the key findings, particularly as the management of advance decisions to refuse treatment of injuries and illnesses following suicidal behaviour is challenging for clinicians[8] and there is a lack of consistency of practice. A review of the literature will be important to inform guidelines for the management of advance decisions following suicidal behaviour.

Aim

To systematically review and synthesise literature on the treatment and clinical management of patients presenting to hospital with an advance decision to refuse treatment following suicidal behaviour without a chronic or terminal physical illness. The review was conducted by researchers in the UK, but an examination of all the existing literature was conducted without language or country restrictions.

For peer review only - http://bmjopen.bmj.com/site/about/guidelines.xhtml

Method

The review was conducted in accordance with PRISMA guidelines[16, 17] and guidance for conducting narrative synthesis in healthcare.[18] There is no protocol for the review. We used the PRISMA checklist when writing our report.[16]

Search strategy and data sources

An initial scoping of the literature was conducted at inception of the study and the findings were used to inform the search strategy. Content experts and clinical practitioners on the research team assisted with compiling key words and/or phrases (see Table 1). In order to take an inclusive approach and enable inclusion of any papers that involved discussion of management of advance decisions following "suicidal behaviour" we included a variety of key search terms relating to non-accidental injury and suicidal attempts. An electronic search of six databases (EMBASE, MEDLINE, PSYCHINFO, Social Policy and Practice, CINAHL and Medline) was conducted, as well as a full electronic search on WestLaw (an online library of UK legal information) using the following search terms: *advance decisions, advance directives* AND *wills, suicide*. Full search strategy for each database is supplied as supplementary information (Supplementary Information 1). In addition, the reference sections of all included sources were consulted and authors' personal files were also searched to ensure that potentially eligible sources were not omitted. No study design, date or language restrictions were imposed.

Literature searches were conducted during the period April 2016 to July 2018. The specific inclusion and exclusion criteria are detailed in Table 2.

Study selection

Titles and abstracts were screened, with a random sample of 10% of the articles independently screened by another researcher. Additional information was sought where there were any disagreements, which were then resolved through discussion. An acceptable

For peer review only - http://bmjopen.bmj.com/site/about/guidelines.xhtml

concordance rate between the inclusion decisions was predefined as agreement on at least 90% of the articles, which was achieved for screening on title and abstract. Full text screening of the selected articles was conducted by two researchers independently, with full agreement being achieved at this stage.

Data extraction and analysis

A preliminary analysis of the data was conducted.[18] Studies were from a range of disciplines (i.e. general medical, psychiatry, ethical, legal) and involved reviews of clinical cases or fictional scenarios. It was deemed appropriate to conduct a narrative synthesis because this particular approach is useful when synthesising textual findings from diverse literatures.[18] Narrative synthesis was conducted in two phases: 1) a *textual synthesis*, and 2) an adapted *thematic framework analysis*.[19]

First, the *textual synthesis* of the data was conducted by extracting key factual information from each study (country of origin, perspective/discipline, factual or fictional case study) and details of the case studies (age of patient, mental health conditions, nature of suicidal behaviour, resulting injuries/illness, hospital admittance, type of advance decision, when advance decision was written, whether patient was conscious, decision-making processes, rationale for decision, outcome). The information was then summarised and tabulated to map the literature that cited the same clinical case. Information from cases only involving a factual case study (i.e. a real clinical case) was extracted because we were interested in information about actual clinical cases, decision-making process and rationale for decisions made. Thus, information was not extracted from reports that discussed a hypothetical scenario for the textual synthesis. Data extraction and summarisation was completed independently by two researchers using a pre-determined data extraction sheet.

Second, an adapted *thematic framework analysis* approach[19] was used to examine key themes discussed in the selected papers. This involved five stages: initial open coding, indexing, descriptive summaries, charting and tabulation and interpretation. *Initial open coding* generated three general categories representing the most discussed issues across

For peer review only - http://bmjopen.bmj.com/site/about/guidelines.xhtml

the selected articles: 1) key issues with an advance decision relating to suicidal behaviour, 2) challenges in clinical decision-making for advance decisions relating to suicidal behaviour, and 3) recommendations for practice. These three categories were used to index the data and as a framework to extract and summarise data. Extracted data was then used to form *descriptive summaries*. Indexing, extracting and summarising were conducted independently by two researchers. Resulting summaries were compared and discussions were held to clarify any differences. *Charting and tabulation* was conducted by charting the summaries by discipline. In order to explore similarities and differences between disciplines, we distinguished between "general medical" as papers written from a general medical practice or emergency services perspective; "psychiatry" as those written by clinical psychiatrists or from a psychiatry perspective, "Nursing" as those written by practising nurses or research nurses, "Bioethics" as those in ethics sections in journals or written by researchers in medical ethics, "Ethics" as those in ethics journals or written by ethics researchers, and "Legal" as those written from a legal perspective and/or by a legal representative. *Interpretation* of the data was conducted by thematic analysis of the summary charts to highlight the main recurrent and most important themes.[18] Two researchers conducted the thematic analysis independently and then discussed and finalised themes. Saturation of the themes was established when no further themes emerged and themes could not be further collapsed. "Vote counting" was used to identify the frequency with which the themes appeared in the selected papers.[20] In the thematic framework analysis all selected studies were included; those involving a factual case and those involving a fictional case, because both involved discussions of concerns, challenges and rationale for decision making relating to management of an advance decision following suicidal behaviour.

Quality assessment

The papers mostly comprised accounts of clinical cases written by clinicians and ethical or legal experts. The methodology quality and synthesis of case series and case reports tool suggested by Murad and colleagues[21] was used to assess the quality of selected studies. Each study was assessed independently across 4 areas of potential bias: selection, ascertainment, causality and reporting. The tool consisted of 5 items each requiring a binary

For peer review only

response to indicate whether the bias was likely. We considered the quality of the study good when all five criteria were fulfilled, moderate when 4 were fulfilled and poor when 3 or less were fulfilled. The methodological quality of included studies was assessed independently by two reviewers and discussion between them where there was disagreement. We also considered the reflexivity of the author/s, their expertise and how they were involved in the clinical case (for example as a clinician or legal/ethics consultant). Authors of the papers reflected on the management of the clinical case, rationale for decision made and issues relating to advance decisions and suicidal behaviour more generally.

Patient and Public Involvement

An expert by-experience was a co-applicant on the NIHR Programme Grant and actively contributed to the study design and objectives. Patient advisors, carers, and clinicians evaluated the relevance and importance of the research questions for the advance decisions component of the grant and the systematic review. Our interim and final results were presented and evaluated by clinicians, academics, patients, and carers. There was also patient input into our dissemination plan, which includes dissemination to clinicians and the relevant patient community.

Results

Systematic search

Results of the systematic search are displayed in Figure 1. After removal of duplicates, the search returned 634 articles, of which 35 were retained after screening based on title/abstract. Following full text screening, 15 articles were retained for data extraction.

For peer review only

Study Characteristics

Descriptive information about the selected articles is displayed in Table 3. Five of the selected articles were from the UK and the others were from the US (n = 7) or Australia (n = 3). A total of six clinical cases were reviewed across the 15 articles (see Table 3), as seven (47%) of the articles reported the same case (Case A, a well-publicised case of a 26 year old woman who died in the UK). Two of the clinical cases presented fictional scenarios.[2, 22]

Study quality assessment

All 15 studies were assessed for bias using the methodology quality and synthesis of case series and case reports tool suggested by Murad and colleagues.[21] Nine of the selected studies were deemed to have moderate methodologic quality and 6 to have poor quality (see supplementary information 2). The quality assessment is supplied as supplementary information (Supplementary Information 2). None of the studies reported the representativeness or selection process relating to the case report, which impacted on the bias ratings. Although case reports are considered to have increased risk of bias, they have profoundly influenced medical literature and advance knowledge and their use in reviews is considered appropriate where no other higher level evidence is available.[21]

Textual synthesis

Examination of clinical cases discussed in the selected articles

Specific information about clinical cases and decision-making is summarised and charted in Table 4. We only included examination of the factual cases (n = 6) in this part of the analysis, because we were interested in the types of real-world cases and decisions made, rather than an examination of a hypothetical scenario.

Patients discussed in the clinical cases varied in age, ranging from 26-86 years old. All patients were noted as having a diagnosis of depression, some were reported as also having

For peer review only

diagnoses of Post-traumatic stress disorder and personality disorders. The suicide methods used in the cases included self-poisoning (n = 3), gunshot incidents (n =2) and hanging (n =1). All patients were found by other people, except one patient who called an ambulance because they did not want to die alone. Four of the patients were reported to have died; the outcome in one case was not specified.

Treatment was provided in only one of the clinical scenarios.[23] In this case, the patient was a psychiatric inpatient and the advance decision was considered part of the suicide attempt, so the patient's treatment refusal specified in the advance decision document was not adhered to.

The rationale for non-treatment in the clinical cases where the patient died varied and was summarised into the following three reasons:

- Advance decision was followed as a *legally-binding document* after checks showed the information was clear and specific, patient was informed of treatment options, had mental capacity at the time of writing and family were in agreement with the decision for non-treatment (n = 1).[8, 24]
- Physical injuries were severe resulting in *poor prognosis* for the patient and the treatment refusal in the advance decision was used as evidence that the patient would not wish to survive with a life-threatening or severely disabling condition. Where possible, families were also consulted (n = 2).[7, 25]
- *Verbal treatment refusal* was used as the basis for the treatment decision, rather than the advance decision, because the patient was conscious and had mental capacity. Consultation with family was not reported in this case. (n =1).[6, 26, 27, 28-30, 31]

The decision-making process was reported to take considerable time and legal and/or ethical consultation took place in all the reported clinical cases.

Differences in opinions about clinical management and decision-making between emergency department clinicians and psychiatric consultants were reported in some of the

For peer review only - http://bmjopen.bmj.com/site/about/guidelines.xhtml

clinical cases.[23, 25] In those cases, emergency department clinicians gave more weight to the advance decision, suggesting it should be adhered to as a legally binding document and the patient remain untreated. In contrast psychiatrists viewed suicide as a consequence of a distressed state and expressed a preference to avoid adherence with the advance decision and treat the patient. Where such conflict arose this was resolved through consultation with the hospital legal team and/or ethics committee.

Thematic analysis

Five themes arose from the thematic analysis and are presented with their corresponding sub-themes and vote-counts in Table 5. We included accounts of fictional cases in the thematic analysis because here we were interested in opinions, views and perspectives of authors.

Themes

1) Tension between patient autonomy and protecting a vulnerable person

Professional dilemma: promoting patient autonomy vs. providing appropriate care

The management of an advance decision in the context of suicidal behaviour was particularly challenging because it went against healthcare professionals' training to preserve life (i.e. adherence to the advance decision could result in the death of the patient while they could recover if they received treatment for their physical condition). This presented clinicians with a dilemma between promoting patients' autonomy by observing their wishes stated in the advance decision and by providing care that was considered in their best interests (e.g. promoting life).[7, 23, 26, 28, 30]

For peer review only

Societal expectation to protect vulnerable person and prevent suicide

Authors also raised the issue that clinicians not only had a professional interest in protecting a vulnerable person, but there was also a societal expectation that suicide should be prevented.[23, 25, 30]

> "While the right to autonomy is strong, in some circumstances there may be competing rights and interests that are sufficient to override a competent decision to refuse treatment. These may include the state's interests in preventing suicide."[30]

The challenge to clinicians was highlighted by an acknowledgement from some authors that adherence to the advance decision in this context was emotive and would feel like assisting suicide.[24, 30]

2) Appropriateness of advance decisions for suicidal behaviour

Mental health symptoms and suicidal ideation fluctuate

Concerns were expressed about whether an advance decision should apply in the context of suicidal behaviour because of patients' distressed state, the potential for suicidal ideation to fluctuate and for treatment preferences to change in the future.[7, 8, 31, 32]

> "The compelling notion that people will change their minds contradicts the primacy of patient autonomy in the consideration of suicide. This is what distinguishes an impulsive suicide attempt from other informed choices to obtain or refuse medical treatment by patients."[7]

Authors from a psychiatric perspective, in particular, viewed suicidal behaviour as a symptom of a mental health condition that was potentially treatable with psychiatric

For peer review only

care.[25] They also expressed concerns about the capacity of a distressed suicidal person to fully comprehend their decision and consider all treatment options available to them.[2, 24, 25, 32] Therefore, it was suggested by some authors that a higher level of mental capacity may be required at the time of writing the advance decision for clinicians to be confident in following it.[8] However, other authors argued that the advance decision should be considered as part of the suicide attempt and as evidence of distressed/disordered thinking,[8, 23, 27, 28] rather than independently of the attempt, and the treatment refusal in the advance decision document should not be adhered to.

Advance decisions for mental and physical health conditions – are they the same?

The difference between an advance decision for suicidal behaviour and for a physical condition was highlighted across the selected papers.[6, 32] Authors from a legal perspective highlighted that the primary aim of an advance decision relating to a suicide attempt is to end life, whereas an advance decision for a chronic or terminal illness is often concerned with managing pain and avoiding prolonged suffering.[6]

There was also debate about the extent to which mental suffering legitimised suicide.[32] Authors from an ethical perspective argued that, typically, healthcare services may be more sympathetic to "end of life" decisions relating to terminal physical health conditions than mental health conditions, thus mental health patients do not receive the same palliative care options as patients without mental health diagnoses.[24] There was some discussion that it should not be assumed that psychiatric pain is more tolerable than physical pain and that both should be considered as having a similar influence on the patient.[24, 25]

3) Uncertainty about the application of legislation

Confusion and anxiety about litigation

Authors from general medical and psychiatry perspectives expressed confusion about legislation and anxiety about litigation,[2, 23, 30] with one stating that the advance decision

For peer review only

document needed to be '*watertight'* to be considered evidence of the patient's.[25] Authors recommended that clear hospital policies be developed for advance decisions in this particular context to overcome the confusion and anxiety about ligation.[23]

> "In addition to the clinical demands associated with treating a patient with a life-threatening condition, clinicians must do their best to ascertain the patient's capacity for his or her apparent decision, consider the correct ethical course, and navigate through uncharted legal waters."[7]

Authors from the UK and Australia highlighted the difficulties in implementing both mental health and mental capacity legislation when managing advance decisions relating to suicidal behaviour.[27, 29, 30, 31] Clinicians needed to consider whether someone who had attempted suicide was suffering with a mental health condition, for which they should be treated against their will. They also needed to judge whether the person had the capacity to make a decision about their treatment and, if so, that the advance decision could apply following verification checks. Some suggested that application of each legislation model (i.e. mental health or mental capacity), in isolation of the other, could result in different outcomes for the patient.[6] Some authors suggested that the difficulty with balancing mental capacity legislation and mental health legislation could be resolved by developing a single legislation that combines both.[8, 27]

Advance decisions are about more than a simple assessment of capacity

A reliance on judging a person's capacity to make a decision in the context of suicidal behaviour was discussed in detail.[8, 22, 24] The capacity assessment was discussed in relation to when the patient was involved in advance care planning and making the decision to write an advance decision to refuse treatment.[8] Capacity assessment was also discussed in relation to clinicians in an emergency situation, when if the person is considered to have capacity the advance decision can be ignored and they can verbally refuse/accept treatment. While this is an important part of some legislation, particularly in the UK, it was suggested that an assessment of capacity should be supplemented with a

For peer review only - http://bmjopen.bmj.com/site/about/guidelines.xhtml

For peer review only

judgment of the authenticity and durability of the patient's decision (i.e. if the decision had been consistent over time).[22, 26] Authors from a psychiatric perspective, in particular, suggested that advance decisions should be regularly reviewed to ensure that they were up-to-date and continued to reflect the patient's desires and preferences.[26, 27, 28]

4) The length of time needed to consider all the evidence vs. rapid decision-making for treatment

Need to fully consider the totality of evidence

Some authors suggested that the increased length of time taken in this particular context arose from the need to consider contextual factors for the suicidal behaviour,[2, 22, 25] the patient's mental health background[27] and the reason for their decision, alongside the usual validation checks and judgment as to presence of mental capacity at the time of making the advance decision. It was also argued that clinicians should take into account wider factors that may have not been present when the person first wrote the advance decision, such as changes in evidence-base for a particular treatment or scientific advances offering new treatment options that may influence the patient's decision.[22]

However, authors highlighted difficulties with gaining access to such evidence, particularly in emergency situations, further adding to the time taken to make a decision.[31] It was noted that advance decisions were often too specific (e.g. related to a specific illness or injury) or too general (e.g. a general refusal of treatment, rather than refusal of a specific treatment), resulting in ambiguity as to the best course of action for the patient and time consuming investigation.[2,25,28] Some authors highlighted that advance decisions were not useful in emergency settings when rapid decision-making was required.[2] Advance decisions may be more appropriate for patients to express refusals of on-going psychiatric treatment (e.g. Electroconvulsive therapy).

For peer review only

Increased gravity of the clinical decision

Authors argued that the gravity of the clinical decision was increased in this context because the patient could die if the advance decision was adhered to when recovery from mental ill health may be possible.[6, 25] Authors suggested that validation checks in this context may need to be more thorough and authors from a legal perspective argued that, because of the increased gravity of the clinical decision, physicians should seek a consensus about clinical management, whilst providing life-sustaining treatment, creating a time-consuming situation.[7, 31]

5) Importance of seeking support and sharing the decision

Drawing up an advance decision as a collaborative process

Some authors argued that when writing an advance decision, patients should be supported by a healthcare professional to consider all possible treatment options.[2, 22, 23, 27, 29] It was suggested that evidence of mental capacity at the time of writing the advance decision should be provided (e.g. verified and signed by the healthcare professional) which could help with clinical decision-making at a later stage.[22] Authors from all the perspectives stressed the importance of also consulting with a physician at the time of writing the advance decision to ensure that it is both specific and general enough to be helpful and informative in a given medical scenario.[23, 27]

Shared decision making

All authors discussed the need for multi-agency decision-making in relation to the management of advance decisions in the context of suicidal behaviour.[7, 27, 28] Suggestions included that clinicians should consult widely, make use of psychiatric expertise, review the patient's psychiatric history and background and seek legal and/or ethical consultation when considering treatment decisions.

For peer review only - http://bmjopen.bmj.com/site/about/guidelines.xhtml

Discussion

Summary of the findings

A comprehensive systematic review of studies examining the management of advance decisions to refuse treatment following suicidal behaviour was conducted. The findings show a paucity of studies in this specific area. Fifteen relevant studies were identified, of which all were reports of clinical cases. With the exception of two papers that noted fictional clinical cases, the others reported on six real clinical cases. Despite having no language or country restrictions to the search, all the studies were from the US, Australia or UK, which have similar legislation relating to advance care planning and advance decisions to refuse treatment.[2]

There were inconsistent views on practice and rationales for the management of advance decisions. Treatment was provided in only one clinical case where the patient was a psychiatric inpatient and the advance decision was considered part of the suicide attempt.[23] In this case the patient survived and later regretted the suicide attempt. In the other clinical cases, treatment was not provided, but rationale for non-treatment differed. Rationale for treatment varied from feeling that the advance decision was legally binding[8, 24] to using the advance decision as an aide to understand the patients' treatment preferences when there was a poor prognosis or a resulting severely disabling condition.[7, 25]

Conflict between clinicians was reported in some of the reports.[23, 25] In the studies where there were conflicts, there were differences in opinions on treatment between emergency department clinicians and psychiatrists. Consultations with mental health care staff were typically sought when a patient presented with an advance decision following suicidal behaviour. Psychiatrists tended to stress the treatable nature of a mental health condition and that the suicidal behaviour was part of the mental health condition. In contrast, emergency department clinicians argued that the advance decision document was legally binding and expressed anxieties about litigation. These differences in opinion about treatment were overcome through consultations with legal and ethical representatives.

For peer review only

The appropriateness of advance decisions with suicidal behaviour was questioned. The questioning of the appropriateness centred largely around two reasons. First, suicide ideation was considered to fluctuate and people could change their mind about their desire to die.[7, 8, 31, 32] Although suicide has been linked to impulsivity,[33, 34] studies show that not all suicides are impulsive.[35] However, recent studies using ecological momentary assessment have shown that suicide ideation varies over short periods of time (i.e. there are changes between hours and days)[36] and follow up studies with suicide survivors tend to acknowledge that they regret the suicide attempt.[37] Second, outcomes for treatment refusal following suicidal behaviour were noted to be potentially different to those for a terminal physical health condition (i.e. the patient could die when there is potential for recovery in the future). [6, 32]

Authors discussed concerns that management of advance decisions following suicidal behaviour may need to be different and present a unique clinical presentation. Similar to findings in this review, anxieties and confusion about legislation relating to advance decisions is also found in studies examining end of life care.[38] However, what does seem to differ is opinions about adherence to the advance decision to refuse treatment for chronic or terminal conditions and sympathy for assisted suicide in end of life care. Healthcare workers report support for assisted suicide relating to end of life care[39] and frustrations with continuing life-sustaining treatment where withdrawing treatment might be considered in the best interest of the patient when they have a life-threatening condition.[23, 40] Those findings indicate quite a contrast with opinions in this review where the focus was on management of advance decisions following suicidal behaviour and an expression of sympathy with the decision was not found. It will be important in future research to examine these differences further by contrasting views on management of advance decisions to refuse treatment following suicidal behaviour for patients with chronic and/or terminal physical conditions and patients who have mental health conditions without chronic or terminal physical conditions.

For peer review only - http://bmjopen.bmj.com/site/about/guidelines.xhtml

Management of the advance decision was difficult both emotionally and ethically for some clinicians because it challenged their professional training and their desire to protect vulnerable patients from suicide. The competing pressures of respecting a patient's right to autonomy while protecting them from the effects of mental disorder found in the current study are a commonly reported dilemma.[41] There is evidence from the present study that support for the right to autonomy may be more dominant in clinicians from emergency medicine disciplines, with those from a psychiatric background prioritising prevention of suicide. A 'middle ground' between these views may help to provide guidance for clinicians. For example, in English law, courts have acknowledged that while some suicidal individuals may have capacity, the overwhelming likelihood is that capacity is impaired to at least some degree.[41] Suicidal ideation has been associated with disordered and impulsive decision making[33, 34] and evidence indicates that most mental health patients presenting to emergency departments are judged as not having capacity to make a treatment decision.[12]Therefore a higher degree of certainty should be required when assessing capacity with suicidal behaviour and clinicians should err on the side of caution .[8] Another potential resolution to this dilemma, particularly in emergency scenarios, may be to provide 'temporary intervention' to allow time for individuals to be assessed and treatment options to be discussed.[41]

An added pressure for clinicians in the management of advance decisions following suicidal behaviour was that they felt there was a societal expectation that suicide should be prevented. Adhering with the advance decision made by the patient by not treating them, not only was seen to go against their professional training to protect the patient, but it was viewed that this may be considered from a society perspective as unacceptable. The dilemma here is that a clinical decision of non-treatment and adherence with the advance decision might be accepted legally, but not socially. Concerns were expressed that this particular presentation of an advance decision met conditions that warranted overriding patients' autonomy because non-adherence with the advance decisions results in prevention of suicide, maintenance of the integrity of the medical professional and preservation of life.[25]

For peer review only - http://bmjopen.bmj.com/site/about/guidelines.xhtml

Recommendations for practice

Decisions made about advance decisions in the context of suicidal behaviour should be made in full consultation with psychiatric teams and with relevant legal and/or ethical advisers. The results also highlight the importance of allocating sufficient time to consider contextual evidence relating to the suicidal behaviour, the authenticity of the treatment decision and verification of the documentation/decision. Given the gravity and emotive nature of a decision in this context, emergency healthcare workers may need increased support and supervision for such incidents.

Findings indicate that it may be helpful, in this particular context, for an advance decision to be written in consultation with a professional healthcare worker and the patient's family. This practice would also ensure that the patient is supported to consider all treatment options, that the advance decision is specific and detailed enough to be useful in an emergency situation and that patients' capacity at the time of writing the advance decision can be assessed and verified. The advance decision should be regularly reviewed and updated to ensure that it reflects the patient's current treatment decisions.

Strengths and Limitations

A strength of this review is that a broad range of articles from different disciplines were included, thus increasing the generalisability of results. However, there were some potential biases in the literature. First, there was a paucity of evidence: only six clinical cases were reported across the selected articles. There was also a risk of bias from the studies themselves, given that they were reviews of single clinical cases. Second, the articles were focussed on the US, UK and Australia, so may have resulted in bias relating to the specific legislation/ethics of those countries. There may be different views on this topic and its management in countries with different implementation of legislation, so it will be

For peer review only

important for future research to compare findings internationally across a wider range of countries.[42-44] Third, as with any syntheses of qualitative data there was potential for bias to be introduced by the research team at the stages of study identification, data extraction and synthesis. This was minimised in the current study by having two researchers carry out these tasks independently and cross-check the findings.

Future directions

Empirical studies, such as interviews and focus groups with clinicians and patients and/or a national clinical survey are important future priorities. Given that the presentation of an advance decision following suicidal behaviour is rare, case reports are likely to continue to be important sources of information in the future and authors should be mindful to ensure that case reports include details about how information about the case were obtained and how representative it is of other cases in this area. Research examining the prevalence of advance decisions relating to suicidal behaviour could shed light on the frequency of such presentations. Suitable platforms for storing advance decisions could also be explored. For example, some have suggested a web application ('app') could better reflect the dynamic nature of treatment refusal[45] and make updating and reassessment easier.

Conclusion

Current literature on the management of advance decisions and suicidal behaviour centres on detailed accounts of clinical cases and demonstrates variability in practice and the rationale behind clinical decisions. Challenges in managing advance decisions specific to suicidal behaviour were evident and there was some debate about whether advance decisions in the context of suicidal behaviour were appropriate in their current form. Taking time to consider all the evidence when making a decision, consulting fully with mental health clinicians and seeking legal and/or ethical advice may help with some of these

For peer review only

challenges. The support of a relevant healthcare professional at the time of writing the advance decision may also be useful.

For peer review only

References

1. Department of Health. Mental Capacity Act. London: Stationery Office, 2005.
2. Byrne, M. The use of advance directives. The use of advance directives. 2002. Nursing Monograph, 13-16.
3. Campbell LA, Kisely SR. Advance treatment directives for people with severe mental illness. The Cochrane Library. 2009 Jan 1. doi: 10.1002/14651858
4. Swanson JW, Swartz MS, Elbogen EB, Van Dorn RA, Ferron J, Wagner HR, McCauley BJ, Kim M. Facilitated psychiatric advance directives: a randomized trial of an intervention to foster advance treatment planning among persons with severe mental illness. American Journal of Psychiatry. 2006 Nov;163(11):1943-51. doi: 10.1176/ajp.2006.163.11.1943
5. Morriss R, Mudigonda M, Bartlett P, Chopra A, Jones S. National survey and analysis of barriers to the utilisation of the 2005 mental capacity act by people with bipolar disorder in England and Wales. Journal of Mental Health. 2017 Jun 19:1-8. 10.1080/09638237.2017.1340613
6. Dresser, R. Suicide attempts and treatment refusals. 2010. Hastings Centre Report, 40(30), 10-11.
7. Frank, C. How to reconcile advance care directives with attempted suicide. The Colorado Lawyer. 2013;42(7):97-101.
8. Kapur, N, Clements, C, Bateman, N, Foex, B, Mackway-Jones, K, Huxtable, R, Gunnell, D, Hawton, K. Advance directives and suicidal behaviour. British Medical Journal. 2010; 341, 590-91.
9. Kapur N, Cooper J, O'Connor RC, Hawton K. Non-suicidal self-injury v. attempted suicide: new diagnosis or false dichotomy? British Journal of Psychiatry. 2013;202(5):326-8. doi: 10.1192/bjp.bp.112.116111. PubMed PMID: WOS:000318839900004.
10. Hawton K, Bergen H, Casey D, Simkin S, Palmer B, Cooper J, Kapur N, Horrocks J, House A, Lilley R, Noble R. Self-harm in England: a tale of three cities. Social psychiatry and psychiatric epidemiology. 2007 Jul 1;42(7):513-21. doi: 10.1007/s00127-007-0199-7
11. Carroll R, Metcalfe C, Gunnell D. Hospital presenting self-harm and risk of fatal and non-fatal repetition: systematic review and meta-analysis. PLoS One. 2014 Feb 28;9(2):e89944. doi: 10.1371/journal.pone.0089944
12. Jacob R, Clare IC, Holland A, Watson PC, Maimaris C, Gunn M. Self-harm, capacity, and refusal of treatment: implications for emergency medical practice. A prospective observational study. Emergency Medicine Journal. 2005 Nov 1;22(11):799-802. doi: 10.1136/emj.2004.018671
13. Kapur N, Clements C, Bateman N, Foëx B, Mackway-Jones K, Hawton K, Gunnell D. Self-poisoning suicide deaths in England: could improved medical management contribute to suicide prevention?. QJM: An International Journal of Medicine. 2010 Aug 4;103(10):765-75. doi: 10.1093/qjmed/hcq128
14. Brinkman-Stoppelenburg A, Rietjens JA, van der Heide A. The effects of advance care planning on end-of-life care: a systematic review. Palliative medicine. 2014 Sep;28(8):1000-25. doi: 10.1177/0269216314526272
15. Houben CH, Spruit MA, Groenen MT, Wouters EF, Janssen DJ. Efficacy of advance care planning: a systematic review and meta-analysis. Journal of the American

For peer review only - http://bmjopen.bmj.com/site/about/guidelines.xhtml

Medical Directors Association. 2014 Jul 31;15(7):477-89. doi: 10.1016/j.jamda.2014.01.008

16. Moher D, Shamseer L, Clarke M, Ghersi D, Liberati A, Petticrew M, Shekelle P, Stewart LA. Preferred reporting items for systematic review and meta-analysis protocols (PRISMA-P) 2015 statement. Systematic reviews. 2015 Dec;4(1):1. doi: 10.1186/2046-4053-4-1
17. Shamseer L, Moher D, Clarke M, Ghersi D, Liberati A, Petticrew M, Shekelle P, Stewart LA. Preferred reporting items for systematic review and meta-analysis protocols (PRISMA-P) 2015: elaboration and explanation. Bmj. 2015 Jan 2;349:g7647. doi. 10.1136/BMJ.g7647.
18. Centre for reviews and dissemination, Undertaking systematic reviews of research on effectiveness, CRD's guidance for carrying out or commissioning reviews, 2009, York, University of York, 3
19. Spencer L, Ritchie J. Qualitative data analysis for applied policy research. In Analyzing qualitative data 2002 Sep 9 (pp. 187-208). Routledge.
20. Ryan R; Cochrane Consumers and Communication Review Group. Cochrane Consumers and Communication Review Group: data synthesis and analysis'. http://cccrg.cochrane.org, June 2013 (accessed 25th April 2018).
21. Murad MH, Sultan S, Haffar S, et al. Methodological quality and synthesis of case series and case reports BMJ Evidence-Based Medicine Published Online First: 02 February 2018. doi: 10.1136/bmjebm-2017-110853
22. Mitchell, M. An analysis of common arguments against advance directives. 2011. Nursing Ethics, 19 (2) 245-51.
23. Cook R, Pan P, Silverman R, Soltys SM. Do-not-resuscitate orders in suicidal patients: Clinical, ethical, and legal dilemmas. Psychosomatics: Journal of Consultation and Liaison Psychiatry. 2010;51(4):277-82.
24. Sontheimer D. Suicide by advance directive? Journal of Medical Ethics. 2008;34(9):e4-e 1p.
25. Chalfin DB, Crippen D, Franklin C, Kelly DF, Kilcullen JK, Streat S, et al. 'Round-table' ethical debate: Is a suicide note an authoritative 'living will'? Critical Care. 2001;5(3):115-24.
26. Callaghan, S. & Ryan, C. J. Refusing medical treatment after attempted suicide: Rethinking capacity and coercive treatment in light of the Kerrie Wooltorton case. Journal of Law and Medicine. 2011. 18 (4) 811-819
27. David A, Hotpur, M, Mora, P, Owen, G, Szmukler, G, Richardson, G. Mentally disordered or lacking capacity? Lessons for managing serious deliberate self-harm: Advance directives and suicidal behaviour. British Medical Journal. 2010;341, 587-589.
28. Muzaffar S. 'To treat or not to treat'. Kerrie Wooltorton, lessons to learn. Emergency Medicine Journal. 2011;28(9):741-4.
29. Richardson, G. Mental capacity in the shadow of suicide: What can the law do? International Journal of Law in Context, 2013, 9 (1), 87-105.
30. Ryan, C., J. & Callaghan, S. C. Legal and ethical aspects of refusing medical treatment after a suicide attempt: the Wooltorton case in the Australian context. Clinical Practice. 2010: 193 (4) 239-243.

For peer review only

31. Szawarski P. Classic cases revisited: The suicide of Kerrie Wooltorton, The Intensive Care. 14: e4
32. Volpe, R. L., Levi, B. H., Blackall, G. F., & Green, M. J. 2012. Exploring the limits of autonomy. Hastings Centre Report, 16-18.
33. Verdier R, Castelnau D, Malafosse A, Courtet P. Impaired decision making in suicide attempters. American Journal of Psychiatry. 2005 Feb 1;162(2):304-10.
34. Dombrovski AY, Hallquist MN. The decision neuroscience perspective on suicidal behavior: evidence and hypotheses. Current opinion in psychiatry. 2017 Jan;30(1):7.
35. Baca–Garcia E, Diaz–Sastre C, Resa EG, Blasco H, Conesa DB, Oquendo MA, Saiz-Ruiz J, De Leon J. Suicide attempts and impulsivity. European archives of psychiatry and clinical neuroscience. 2005 Apr 1;255(2):152-6
36. Kleiman EM, Turner BJ, Fedor S, Beale EE, Huffman JC, Nock MK. Examination of real-time fluctuations in suicidal ideation and its risk factors: Results from two ecological momentary assessment studies. Journal of abnormal psychology. 2017 Aug;126(6):726.
37. Rosen DH. Suicide survivors: a follow-up study of persons who survived jumping from the Golden Gate and San Francisco-Oakland Bay Bridges. Western Journal of Medicine. 1975 Apr;122(4):289.
38. Meisel A, Snyder L, Quill T, American College of Physicians-American Society of Internal Medicine End-of-Life Care Consensus Panel. Seven legal barriers to end-of-life care: myths, realities, and grains of truth. Jama. 2000 Nov 15;284(19):2495-501.
39. Scherer Y, Jezewski MA, Graves B, Wu YW, Bu X. Advance directives and end-of-life decision making survey of critical care nurses' knowledge, attitude, and experience. Critical Care Nurse. 2006 Aug 1;26(4):30-40.
40. Solomon MZ, O'Donnell L, Jennings B, Guilfoy V, Wolf SM, Nolan K, Jackson R, Koch-Weser D, Donnelley S. Decisions near the end of life: professional views on life-sustaining treatments. American Journal of Public Health. 1993 Jan;83(1):14-23.
41. Allen N. The right to life in a suicidal state. International journal of law and psychiatry. 2013 Sep 1;36(5-6):350-7. doi: 10.1016/j.ijlp.2013.09.002Blank RH. End-of-life decision making across cultures. The Journal of Law, Medicine & Ethics. 2011 Jun 1;39(2):201-14. doi: 10.1111/j.1748-720X.2011.00589.x
42. Blank RH. End-of-life decision making across cultures. The Journal of Law, Medicine & Ethics. 2011 Jun 1;39(2):201-14. doi: 10.1111/j.1748-720X.2011.00589.x
43. Mishara BL, Weisstub DN. The legal status of suicide: A global review. International journal of law and psychiatry. 2016 Jan 1;44:54-74. doi: 10.1016/j.ijlp.2015.08.032
44. Van Wijmen MP, Rurup ML, Pasman HR, Kaspers PJ, Onwuteaka-Philipsen BD. Advance directives in the Netherlands: An empirical contribution to the exploration of a cross-cultural perspective on advance directives. Bioethics. 2010 Mar 1;24(3):118-26. doi: 10.1111/j.1467-8519.2009.01788.x
45. Huxtable R. Advance decisions: worth the paper they are (not) written on?. End of Life Journal. 2015 Sep 1;5(1):e000002.doi:10.1136/eoljnl-2015-000002

For peer review only

Table 1. Search terms for each topic

Advance directives	*OR*	Mental capacity	*AND*	Suicidal behaviour
advance decisions		mental		Suicide
advance directives		competency		attempted suicide
advance statement		mental capacity		self-mutilation
living will(s)				self-harm
mental health directive				deliberate self-harm
Ulysses contract(s)				parasuicide
psychiatric will(s)				self-injurious behaviour
antecedent decision/wish				drug overdose
pre-emptive suicide				self-immolation
antecedent refusal				self-poisoning
resuscitation order				self-destructive behaviour
health care power of attorney				auto aggression
				automutilation

For peer review only

Table 2. PICOS criteria for inclusion and exclusion

Parameter	Inclusion criteria	Exclusion criteria
Patients	Patients over 18 years who present to hospital with advance decisions* (also include Do Not Resuscitate orders, DNRs) following suicidal behaviour (including attempted suicide, deliberate self-harm, self-injurious behaviour, drug overdose, self-poisoning, self-destructive behaviour) with no existing chronic or terminal physical conditions	Patients who present to hospital with advance decisions but with primary conditions which were not mental health related e.g. HIV/AIDS, chronic physical health conditions or disabilities, neurodegenerative diseases and/or specific patient groups e.g. mother/baby.
Intervention	medical management and/or medico legal and/or ethical consultation/discussion	medical management of euthanasia, assisted suicide, end of life, wills/inheritance (i.e. monetary or property issues)
Comparator		
Outcomes	Adherence/non-adherence with advance decision, treatment, patient outcome (i.e. death)	
Study design	Opinion and review articles, Case studies, Empirical studies/surveys	Book reviews, Reponses to articles, conference abstracts

** or other terms such as advance decisions, advance directives, advance statement, living will(s), mental health directive, Ulysses contract(s), psychiatric will(s), mental competency, mental capacity, health care power of attorney, antecedent decision/wish, pre-emptive suicide, antecedent refusal, resuscitation order or living will, advance directive, Ulysses contract*

For peer review only

Table 3. Description of selected studies

Author	Date	Country	Perspective#	Fictional/ Factual case	Case reported*
Bryne[2]	2002	Australia	Nursing	Fictional	--
Callaghan & Ryan[26]	2011	Australia	Bioethics	Factual	A
Chalfin et al[25]	2001	US, Philadelphia, New York, New Zealand	Emergency & Acute medicine/ Bioethics	Factual	B
Cook et al[23]	2010	US, Illinois	Psychiatry	Factual	C
Dresser[6]	2010	US, New York	Legal	Factual	A
David et al[27]	2010	UK	Psychiatry	Factual	A
Frank[7]	2013	US, Colorado	Legal	Factual	D
Kapur et al[8]	2010	UK	Psychiatry	Factual	E
Mitchell[22]	2011	US, San Diego	Ethical	Fictional	--
Muzaffer[28]	2011	UK	Psychiatry	Factual	A
Richardson[29]	2013	UK	Legal	Factual	A
Ryan & Callaghan[30]	2010	Australia	Psychiatry	Factual	A
Sontheimer[24]	2008	US, Springfield	Bioethics	Factual	E
Szawarski[31]	2013	UK	Bioethics	Factual	A
Volpe et al[32]	2012	US, New York	Bioethics	Factual	F

Note: *For specific details about each case see Table 4, note fictional cases have not been given a case report ID
#where the perspective is not clearly stated this has been derived from the author(s) background and professional experience

For peer review only

Table 4. Description of clinical cases discussed in selected studies

Case	Reference	Age	Mental health conditions	Nature of SA	Resulting Injuries/ illness	Hospital admittance	Nature of the AD	When written?	Patient conscious ?	Decision Making process	Rationale for decision	Outcome
A	6, 26, 27, 28-30, 31	26	Depression generalised anxiety disorder, PTSD, BPD	Self-poisoning (anti-freeze)	Not stated	Presented herself at hospital	Letter	3 days prior	Yes	Medical staff discussed the patient's mental capacity and sought legal advice.	The patient's wishes were clear in the letter but the patient was conscious, judged to have capacity and refusing treatment.	Death
B	25	46	Severe depression	Gunshot to face	Pain and severe facial injury	Gunshot reported by neighbours	Suicide note	not stated	Yes (not coherent)	The attending physicians thought life-support should be removed as the patient's "will" was clear and authoritative. The psychiatrist thought suicide was pathological and the condition was treatable so the patient should be treated. Clinicians consulted widely and sought legal advice	The suicide note was accepted as a living will. The patient had a desire to die due to psychological pain. The suicide attempt left the patient in a severely disabled state.	Death
C	23	57	Depression generalised anxiety disorder, PTSD, BPD	Self-poisoning (opiates)	Respiratory distress	Psychiatric inpatient	DNR	Prior to inpatient admittance	Not stated	There was conflict between clinicians; the psychiatrist argued that the DNR should not be followed because it was a suicide attempt. The legal/ethics committee was consulted who supported continued treatment.	DNR considered an effort to prepare for a suicide attempt and should not be honoured.	Survived and regretted the suicide attempt.
D	7	35	Depression and drug abuse	Hanging	Brain injury	Found by family	AD	Not stated	No	There were concerns that adherence to the AD would result in the patient's death. Clinicians sought legal advice.	The patient had poor prognosis and the family gave consent for clinicians to stop treatment.	Death
E	8, 24	52	Depression generalised anxiety disorder, PTSD, BPD	Self-poisoning (insulin)	Coma	Found at home	AD	2 years prior	No	The AD mentioned no treatment for a terminal condition. The patient was not in a terminal condition and there were concerns that injury was the result of a suicide attempt and whether the AD should be adhered to in a suicidal context. Approached family and held an ethics committee consultation.	The patient's wishes were judged to be clear, the patient was considered to be informed about treatment options and had mental capacity at the time of writing the AD and the family were in agreement.	Death
F	32	86	Not stated	Gunshot to chest	Damage to pancreas and colon	Not stated	AD	Not stated	Yes (not always coherent)	Medical team argue that the nature in which the physical condition was caused (i.e. suicidal behaviour) should impact on treatment	Not stated	Not stated

Note: *for details about articles see Table 3, SA = suicide attempt, AD = advance directive, PTSD = post-traumatic stress disorder, BPD = borderline personality disorder

For peer review only

Table 5. Themes from the selected articles

Theme	Sub-themes	Theme Descriptor	Perspectives	References	Count %
Tension between patient autonomy and protecting a vulnerable person	*Professional dilemma: promoting patient autonomy vs. providing appropriate care*<br><br>*Societal expectation to protect vulnerable person and prevent suicide*	Tension between acting in accordance with patients' wishes for their medical treatment while promoting their best interests presented clinicians with a professional ethical dilemma. Clinicians also had a personal ethical dilemma, as there is societal pressure to protect vulnerable people and prevent suicide.	Psychiatry, Bioethics, Legal	7, 22, 24, 27, 29	5 (33%)
Appropriateness of advance decisions for suicidal behaviour	*Mental health symptoms and suicidal ideation fluctuate*<br><br>*Advance decisions for mental and physical health conditions – are they the same?*	There were questions about whether an advance decision "fits" in relation to suicide without an existing physical illness because mental state, mental health and suicide ideation fluctuate. Such scenarios are different from decisions made about treatment for a chronic or terminal physical condition.	Medical, Psychiatry, Bioethics, Legal	2, 6-8, 23-25, 27, 29-32	12 (80%)
Uncertainty about the application of legislation	*Confusion and anxiety about litigation*<br><br>*Advance decisions are about more than a simple assessment of capacity*	Legislation around advance decisions was seen as confusing and there was anxiety about ligation. It was noted that mental capacity legislation overlapped with mental health legislation and policy. There were concerns that relying on a capacity decision was not sufficient and the authenticity of the advance decision needed to be considered	Medical, Psychiatry, Bioethics, Legal	2, 8, 22-29, 31	11 (73%)
The length of time needed to consider all the evidence vs. rapid decision-making for treatment	*Need to fully consider the totality of evidence*<br><br>*Increased gravity of the clinical decision*	Clinical decisions were considered to be complex, involving an assessment of mental capacity, verification of the advance decision, and consideration of contextual factors. Therefore sufficient time was needed in which to consider all of the evidence.	Medical, Psychiatry, Bioethics Legal	2, 8, 25-27	5 (33%)
Importance of seeking support and sharing the decision	*Drawing up an advance decision as a collaborative process*<br><br>*Shared decision making*	Sharing the decision-making and seeking support, both at the time of writing the advance decision and when treating the patient, was viewed as important.	Medical, Psychiatry, Bioethics, Legal	2, 7, 24-28, 30, 31	9 (60%)

For peer review only - http://bmjopen.bmj.com/site/about/guidelines.xhtml

Figures

Figure 1. Flow chart of results from initial search

For peer review only

For peer review only - http://bmjopen.bmj.com/site/about/guidelines.xhtml

Figure 1. Flow chart of results from initial search

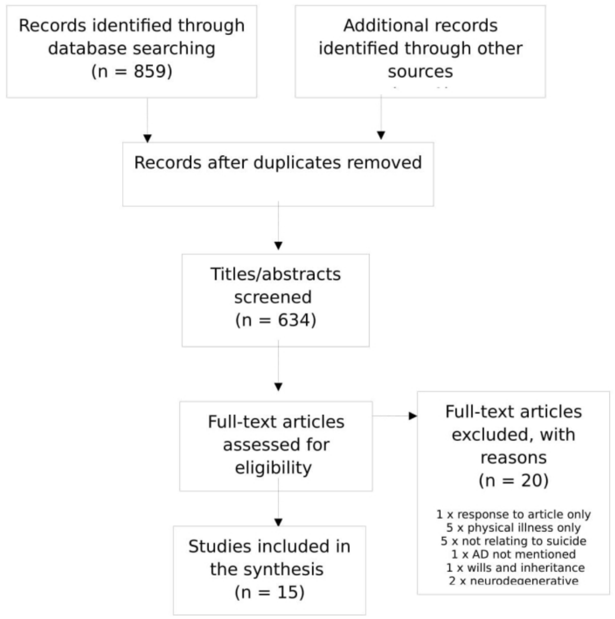

Figure 1

139x198mm (300 x 300 DPI)

Supplementary Information 1: Database Search Strategy

Psychinfo:

(((advance decision or advance directive or advance statement or living will or living wills or mental health directive or Ulysses contract or Ulysses contracts or psychiatric will or psychiatric wills or mental competency or mental capacity or healthcare power of attorney or health care power of attorney or antecedent decision or antecedent wish* or preemptive suicide or antecedent refusal or resuscitation orders)

and

(suicide or attempted suicide or self mutilation or self-harm or deliberate self-harm or self harm or parasuicid$ or para-suicd$ or "self-injurious behaviour" or drug overdose or self immolation or self poisoning or self-destructive behav or autoaggress$ or automutilia$)) not (euthanasia or assisted suicide)).af.

Pubmed:

(“advance decisions” or “advance directives” or “advance statement” or “living will” or “living wills” or “mental health directive” or “ulysses contract” or “ulysses contracts” or “psychiatric will” or “psychiatric wills” or “mental competency” or “mental capacity” or “healthcare power attorney” or “healthcare power of attorney” or “antecedent decision” or “antecedent wish” or “preemptive suicide” or “antecedent refusal” or “resuscitation orders” or “do not resuscitate” or DNR order)
suicid

EBESCO:

(suicide or attempted suicide or self mutilation or self-harm or deliberate self-harm or self harm or parasuicid$ or para-suicd$ or "self-injurious behaviour" or drug overdose or self immolation or self poisoning or self-destructive behav or autoaggress$ or automutilia$).ab

and

(advance decision or advance directive or advance statement or living will or living wills or mental health directive or Ulysses contract or Ulysses contracts or psychiatric will or psychiatric wills or mental competency or mental capacity or healthcare power of attorney or health care power of attorney or antecedent decision or antecedent wish* or preemptive suicide or antecedent refusal or resuscitation orders).ab

For peer review only

EMBASE:

(advance decision or advance directive or advance statement or living will or living wills or mental health directive or Ulysses contract or Ulysses contracts or psychiatric will or psychiatric wills or mental competency or mental capacity or healthcare power of attorney or health care power of attorney or antecedent decision or antecedent wish* or preemptive suicide or antecedent refusal or resuscitation orders).ab.

and

(suicide or attempted suicide or self mutilation or self-harm or deliberate self-harm or self harm or parasuicid$ or para-suicd$ or "self-injurious behaviour" or drug overdose or self immolation or self poisoning or self-destructive behav or autoaggress$ or automutilia$).ab

MEDLINE:

(((advance decision or advance directive or advance statement or living will or living wills or mental health directive or Ulysses contract or Ulysses contracts or psychiatric will or psychiatric wills or mental competency or mental capacity or healthcare power of attorney or health care power of attorney or antecedent decision or antecedent wish* or preemptive suicide or antecedent refusal or resuscitation orders) and (suicide or attempted suicide or self mutilation or self-harm or deliberate self-harm or self harm or parasuicid$ or para-suicd$ or "self-injurious behaviour" or drug overdose or self immolation or self poisoning or self-destructive behav or autoaggress$ or automutilia$)) not (euthanasia and assisted suicide)).ab.

CINAHL

AB (advance decisions OR (advance directives and living wills) OR mental capacity OR mental competency OR health care power of attorney OR antecedent decision OR preemptive suicide OR resuscitation orders OR (dnr or do not resuscitate) OR (dnr orders and ethical principles))

AND AB suicide OR suicide attempt OR self-harm OR self harm OR deliberate self harm OR self-injurious behavior OR (self injury or self harm or self mutilation) OR drug overdose OR self immolation OR self-destructiv behaviors OR self-poisoning
AB ((advance decisions OR (advance directives and living wills) OR mental capacity OR mental competency OR health care power of attorney OR antecedent decision OR preemptive suicide OR resuscitation orders OR (dnr or do not resuscitate) OR (dnr orders and ethical principles))) AND AB (suicide OR suicide attempt OR self-harm OR self harm OR deliberate self harm OR self-injurious behavior OR (self injury or self harm or self mutilation) OR drug overdose OR self immolation OR self-destructiv behaviors OR self-poisoning) NOT AB assisted suicide NOT AB (euthanasia or assisted suicide)

For peer review only

Social Policy and Practice:

(("advance decisions" or "advance directives" or "advance statement" or "living will" or "living wills" or "mental health directive" or "ulysses contract" or "ulysses contracts" or "psychiatric will" or "psychiatric wills" or "mental competency" or "healthcare power attorney" or "healthcare power of attorney" or "antecedent decision" or "antecedent wish" or "pre emptive suicide" or "preemptive suicide" or "antecedent refusal" or "resuscitation orders" or "do not resuscitate" or "DNR order") not (euthanasia and "assisted suicide")).af.

and

(suicide or "attempted suicide" or "self-mutilation" or "deliberate self-harm" or "self-harm" or Parasuicide or Suicid* or "drug-overdose" or "self-poisioning" or "self-immolation" or "suicidal behav*" or "self-destructive behav*" or Autoaggress$ or "self-injurious behav*" or "non suicidal self-injury" or "non fatal self-harm" or "completed suicide" or automutilla$).af

For peer review only

Supplementary Information 2: Quality Assessment of studies

	Question 1		Question 2		Question 3		Question 4		Question 5		Methodological quality
Author	Yes	No	Yes	No	Yes	No	Yes	No	Yes	No	
Bryne[2]		X		X		X		X		X	Poor
Callaghan & Ryan[22]		X	X		X		X		X		Moderate
Chalfin et al[23]		X	X			X	X		X		Poor
Cook et al[24]		X	X		X		X		X		Moderate
Dresser[6]		X		X		X	X			X	Poor
David et al[25]		X	X		X		X		X		Moderate
Frank[7]		X	X			X		X	X		Poor
Kapur et al[8]		X	X		X			X		X	Poor
Mitchell[26]		X		X		X		X		X	Poor
Muzaffer[27]		X	X			X	X			X	Poor
Richardson[28]		X	X		X		X		X		Moderate
Ryan & Callaghan[29]		X	X		X		X		X		Moderate
Sontheimer[30]		X	X		X			X	X		Poor
Szawarski[31]		X	X		X		X		X		Moderate
Volpe et al[32]		X	X			X		X		X	Poor

Note: *Selection*: question 1: Did the patient(s) represent the whole experience of the investigator or is the selection method unclear to the extent that other patients with similar presentations may not have been presented?; *Ascertainment*: question 2: Was the case adequately ascertained?, question 3: Was the outcome adequately ascertained?; *Causality*: question 4: Was follow-up long enough for outcomes to occur?; *Reporting*: question 5: Is the case described with sufficient details to allow practitioners to make inferences on their own practice?

For peer review only

For peer review only - http://bmjopen.bmj.com/site/about/guidelines.xhtml

PRISMA 2009 Checklist

Section/topic	#	Checklist item	Reported on page #
**TITLE**			
Title	1	Identify the report as a systematic review, meta-analysis, or both.	1
**ABSTRACT**			
Structured summary	2	Provide a structured summary including, as applicable: background; objectives; data sources; study eligibility criteria, participants, and interventions; study appraisal and synthesis methods; results; limitations; conclusions and implications of key findings; systematic review registration number.	4
**INTRODUCTION**			
Rationale	3	Describe the rationale for the review in the context of what is already known.	7
Objectives	4	Provide an explicit statement of questions being addressed with reference to participants, interventions, comparisons, outcomes, and study design (PICOS).	7
**METHODS**			
Protocol and registration	5	Indicate if a review protocol exists, if and where it can be accessed (e.g., Web address), and, if available, provide registration information including registration number.	No protocol
Eligibility criteria	6	Specify study characteristics (e.g., PICOS, length of follow-up) and report characteristics (e.g., years considered, language, publication status) used as criteria for eligibility, giving rationale.	8 (Table 1 and 2)
Information sources	7	Describe all information sources (e.g., databases with dates of coverage, contact with study authors to identify additional studies) in the search and date last searched.	8
Search	8	Present full electronic search strategy for at least one database, including any limits used, such that it could be repeated.	8 (Table 1 and 2)
Study selection	9	State the process for selecting studies (i.e., screening, eligibility, included in systematic review, and, if applicable, included in the meta-analysis).	8 (Table 1 and 2)
Data collection process	10	Describe method of data extraction from reports (e.g., piloted forms, independently, in duplicate) and any processes for obtaining and confirming data from investigators.	9
Data items	11	List and define all variables for which data were sought (e.g., PICOS, funding sources) and any assumptions and simplifications made.	9
Risk of bias in individual studies	12	Describe methods used for assessing risk of bias of individual studies (including specification of whether this was done at the study or outcome level), and how this information is to be used in any data synthesis.	10
Summary measures	13	State the principal summary measures (e.g., risk ratio, difference in means).	n/a

For peer review only

PRISMA 2009 Checklist

Synthesis of results	14	Describe the methods of handling data and combining results of studies, if done, including measures of consistency (e.g., I^2) for each meta-analysis.	9

Section/topic	#	Checklist item	Reported on page #
Risk of bias across studies	15	Specify any assessment of risk of bias that may affect the cumulative evidence (e.g., publication bias, selective reporting within studies).	10
Additional analyses	16	Describe methods of additional analyses (e.g., sensitivity or subgroup analyses, meta-regression), if done, indicating which were pre-specified.	9
**RESULTS**			
Study selection	17	Give numbers of studies screened, assessed for eligibility, and included in the review, with reasons for exclusions at each stage, ideally with a flow diagram.	10 (Figure 1)
Study characteristics	18	For each study, present characteristics for which data were extracted (e.g., study size, PICOS, follow-up period) and provide the citations.	10-12, 21 (Table 3)
Risk of bias within studies	19	Present data on risk of bias of each study and, if available, any outcome level assessment (see item 12).	n/a
Results of individual studies	20	For all outcomes considered (benefits or harms), present, for each study: (a) simple summary data for each intervention group (b) effect estimates and confidence intervals, ideally with a forest plot.	Textual analysis 10-12
Synthesis of results	21	Present results of each meta-analysis done, including confidence intervals and measures of consistency.	Synthesis of Qual results 12-17
Risk of bias across studies	22	Present results of any assessment of risk of bias across studies (see Item 15).	Noted in discussion p19
Additional analysis	23	Give results of additional analyses, if done (e.g., sensitivity or subgroup analyses, meta-regression [see Item 16]).	n/a
**DISCUSSION**			
Summary of evidence	24	Summarize the main findings including the strength of evidence for each main outcome; consider their relevance to key groups (e.g., healthcare providers, users, and policy makers).	17-18
Limitations	25	Discuss limitations at study and outcome level (e.g., risk of bias), and at review-level (e.g., incomplete retrieval of identified research, reporting bias).	19

For peer review only

PRISMA 2009 Checklist

Conclusions	26	Provide a general interpretation of the results in the context of other evidence, and implications for future research.	20
**FUNDING**			
Funding	27	Describe sources of funding for the systematic review and other support (e.g., supply of data); role of funders for the systematic review.	2

From: Moher D, Liberati A, Tetzlaff J, Altman DG, The PRISMA Group (2009). Preferred Reporting Items for Systematic Reviews and Meta-Analyses: The PRISMA Statement. PLoS Med 6(7): e1000097. doi:10.1371/journal.pmed1000097

For more information, visit: **www.prisma-statement.org**.

Page 2 of 2

For peer review only

ENTREQ checklist (Enhancing transparency in reporting the synthesis of qualitative research) *

No. Item	Guide questions/description	Reported on Page #
1. Aim	State the research question the synthesis addresses	8
2. Synthesis methodology	Identify the synthesis methodology or theoretical framework which underpins the synthesis, and describe the rationale for choice of methodology (e.g. meta-ethnography, thematic synthesis, critical interpretive synthesis, grounded theory synthesis, realist synthesis, meta-aggregation, meta-study, framework synthesis)	10-11
3. Approach to searching	Indicate whether the search was pre-planned (comprehensive search strategies to seek all available studies) or iterative (to seek all available concepts until they theoretical saturation is achieved)	9
4. Inclusion criteria	Specify the inclusion/exclusion criteria (e.g. in terms of population, language, year limits, type of publication, study type	Table 2
5. Data sources	Describe the information sources used (e.g. electronic databases (MEDLINE, EMBASE, CINAHL, psycINFO), grey literature databases (digital thesis, policy reports), relevant organisational websites, experts, information specialists, generic web searches (Google Scholar) hand searching, reference lists) and when the searches conducted; provide the rationale for using the data sources	9
6. Electronic Search strategy	Describe the literature search (e.g. provide electronic search strategies with population terms, clinical or health topic terms, experiential or social phenomena related terms, filters for qualitative research, and search limits)	9<br>Supplementary Info 1
7. Study screening methods	Describe the process of study screening and sifting (e.g. title, abstract and full text review, number of independent reviewers who screened studies	9-10
8. Study characteristics	Present the characteristics of the included studies (e.g. year of publication, country, population, number of participants, data collection, methodology, analysis, research questions)	12<br>Table 2
9. Study selection results	Identify the number of studies screened and provide reasons for study exclusion (e,g, for comprehensive searching, provide numbers of studies screened and reasons for exclusion indicated in a figure/flowchart; for iterative searching describe reasons for study exclusion and inclusion based on modifications to the research question and/or contribution to theory development)	12-13<br>Figure 1
10. Rationale for appraisal	Describe the rationale and approach used to appraise the included studies or selected findings (e.g. assessment of conduct (validity and robustness), assessment of reporting (transparency), assessment of content and utility of the findings)	11-13
11. Appraisal items	State the tools, frameworks and criteria used to appraise the studies or selected findings (e.g. Existing tools: CASP, QARI, COREQ, Mays and Pope [25]; reviewer developed tools; describe the domains assessed: research team, study design, data analysis and interpretations, reporting)	11-13
12. Appraisal process	Indicate whether the appraisal was conducted independently by more than one reviewer and if consensus was required	13
13. Appraisal	Present results of the quality assessment and indicate which	13,

For peer review only

results	articles, if any, were weighted/excluded based on the assessment and give the rationale	Supplementary info 2
14. Data extraction	Indicate which sections of the primary studies were analysed and how were the data extracted from the primary studies? (e.g. all text under the headings “results /conclusions” were extracted electronically and entered into a computer software)	10-11
15. Software	State the computer software used, if any	n/a
16. Number of reviewers	Identify who was involved in the coding and analysis	10-11
17. Coding	Describe the process for coding of data (e.g. line by line coding to search for concepts)	11
18. Study comparison	Describe how were comparisons made within and across studies (e.g. subsequent studies were coded into pre-existing concepts, and new concepts were created when deemed necessary)	11
19. Derivation of themes	Explain whether the process of deriving the themes or constructs was inductive or deductive	11
20. Quotations	Provide quotations from the primary studies to illustrate themes/constructs, and identify whether the quotations were participant quotations of the author’s interpretation	15-20
21. Synthesis output	Present rich, compelling and useful results that go beyond a summary of the primary studies (e.g. new interpretation, models of evidence, conceptual models, analytical framework, development of a new theory or construct)	13-20

* Reference: Tong A, Flemming K, McInnes E, Oliver SA, Craig J. Enhancing transparency in reporting the synthesis of qualitative research: ENTREQ. BMC Medical Research Methodology 2012, 12:181.

For peer review only
